# Ambulatory Care vs Overnight Hospitalization After Anterior Surgery for Cervical Radiculopathy: The FACADE Randomized Clinical Trial

**DOI:** 10.1001/jamanetworkopen.2024.47459

**Published:** 2024-11-27

**Authors:** Kimmo Lönnrot, Simo Taimela, Jarno Satopää, Ilkka Saarenpää, Ville Leinonen, Juri Kivelev, Marja Silvasti-Lundell, Johannes Förster, Mikko Pitkänen, Rahul Raj, Mikko Kauppinen, Riitta Westermarck, Behnam Rezai Jahromi, Anniina Koski-Palkén, Matti Seppälä, Leena Kivipelto, Jussi Antinheimo, Miikka Korja, Tomasz Czuba, Teppo L. N. Järvinen

**Affiliations:** 1 Department of Neurosurgery, University of Helsinki and Helsinki University Hospital, Helsinki, Finland; 2 Finnish Centre for Evidence-Based Orthopaedics, University of Helsinki, Helsinki, Finland; 3 Department of Orthopaedics and Traumatology, University of Helsinki and Helsinki University Hospital, Helsinki, Finland; 4 Neurocenter, Department of Neurosurgery, Turku University Hospital and University of Turku, Turku, Finland; 5 Department of Neurosurgery, Kuopio University Hospital, Kuopio, Finland; 6 Institute of Clinical Medicine, University of Eastern Finland, Kuopio, Finland; 7 Department of Anesthesiology, Intensive Care and Pain Medicine, Helsinki University Hospital, Helsinki, Finland; 8 Department of Anesthesia, Orthopedic Hospital Orton, Helsinki, Finland; 9 Research Unit of Clinical Neuroscience, University of Oulu, Oulu, Finland; 10 Department of Neurosurgery, Oulu University Hospital, Oulu, Finland; 11 Division of Perioperative Services, Intensive Care Medicine and Pain Management, Turku University Hospital and University of Turku, Turku, Finland; 12 Department of Anesthesiology and Intensive Care, University of Turku, Turku, Finland; 13 Department of Clinical Sciences, Orthopaedics, Lund University, Lund, Sweden; 14 Department of Molecular and Clinical Medicine, University of Gothenburg, Gothenburg, Sweden

## Abstract

**Importance:**

During the past decade, spinal surgical procedures have been increasingly performed on an ambulatory basis, a change in clinical practice supported only by observational evidence thus far. There have been no randomized clinical trials (RCTs) comparing ambulatory care with inpatient care after any spinal surgery.

**Objective:**

To assess whether ambulatory care is noninferior to overnight hospital surveillance in functional outcome, as measured by Neck Disability Index (NDI), in adults after anterior cervical decompression and fusion (ACDF) for cervical radiculopathy.

**Design, Setting, and Participants:**

This parallel group, noninferiority RCT enrolled patients at 3 tertiary neurosurgical centers in Finland (University Hospitals of Helsinki, Turku, and Oulu) between June 2019 and February 2021, with a final follow-up in October 2021. Patients aged 18 to 62 years who underwent 1-level or 2-level ACDF due to cervical radiculopathy were assessed for eligibility. After surgery, participants were randomly assigned to either ambulatory care or overnight hospital surveillance. Intention-to-treat data analyses were conducted in August 2022.

**Interventions:**

Patients randomly assigned to ambulatory care were kept under surveillance for 6 to 8 hours after surgery and then discharged. Patients assigned to overnight hospital surveillance were kept in the ward for 24 hours or longer.

**Main Outcomes and Measures:**

The primary outcome was NDI at 6 months. A minimal important difference of 17.3 percentage points for NDI was set as the margin of noninferiority.

**Results:**

Among the 105 patients (mean [SD] age, 47.0 [7.9] years; 54 women [50%]) randomly assigned to ambulatory care (n = 52) or overnight hospital surveillance (n = 53), 94 (90%) completed the trial. Four (8%) patients who received ambulatory care crossed over to overnight hospital surveillance. Seventy-nine patients (75%) had a 1-level ACDF and 26 (25%) had a 2-level ACDF. At 6 months after surgery, the mean NDI was 13.3% (95% CI, 9.3%-17.3%) in the ambulatory care group and 12.2% (95% CI, 8.2%-16.2%) in the overnight hospital surveillance group (between-group mean difference, 1.1 [95% CI, −4.6 to 6.8] percentage points).

**Conclusions and Relevance:**

In this RCT comparing functional outcomes of ambulatory care vs overnight hospital surveillance after ACDF, ambulatory care resulted in noninferior functional outcomes.

**Trial Registration:**

ClinicalTrials.gov Identifier: NCT03979443

## Key Points

**Question:**

Does ambulatory care result in noninferior functional outcome compared with overnight hospital surveillance after anterior cervical decompression and fusion (ACDF) in adults with cervical radiculopathy?

**Findings:**

In this randomized clinical trial of 105 patients who underwent ACDF, the postoperative 6-month Neck Disability Index differed by 1.1 percentage points between patients who received ambulatory care vs overnight hospital surveillance.

**Meaning:**

Findings of this trial indicate that among adults who underwent ACDF for cervical radiculopathy, functional outcome at 6 months after surgery was noninferior in the ambulatory care group compared with the overnight hospital surveillance group.

## Teaser

This randomized clinical trial examines whether functional outcomes of overnight hospital surveillance vs ambulatory care after anterior cervical decompression and fusion were noninferior in working adults aged 18 to 62 years in Finland.

## Introduction

Hospital stays are expensive and may not always be in patients’ best interests. Advancements in medical interventions have increasingly enabled a transition from inpatient to outpatient care. Spinal surgery represents a prime example; new treatment protocols and other improvements have allowed a shift from inpatient toward ambulatory care in a growing number of cases.^1^ Accordingly, ambulatory care is being popularized for various spinal procedures,^2,3,4,5,6^ such as in a US-based clinical practice guideline on anterior cervical surgery.^7^

By offering patients the chance to recover at home while possibly reducing costs, the shift toward ambulatory care after spinal surgery has merit as long as evidence shows equivalent outcomes and safety to those of inpatient care.^1^ Thus far, such evidence has been only observational. According to a systematic review of studies in ambulatory spine surgery, no level I or II study has been published in the previous 5 years that compared ambulatory care with inpatient care.^2^

Anterior cervical decompression and fusion (ACDF) is an ideal candidate for such a level I study. Cervical radiculopathy—defined as pain radiating into the shoulder and/or arm with possible muscle weakness—caused by cervical nerve root irritation can be treated by ACDF or posterior cervical foraminotomy.^8^ ACDF is one of the most common spinal surgeries. In 2013, over 132 000 ACDF procedures were performed in the US, with an overall increasing trend.^9^ Yet according to the American College of Surgeons’ National Surgical Quality Improvement Program database,^10^ over 70% of all elective ACDF procedures between 2006 and 2016 in the US were still performed in the inpatient setting.

Observational studies have provided preliminary evidence suggesting equivalent outcomes between ambulatory and inpatient care.^3,4,5,6,11,12,13,14^ However, observational data are prone to various biases. For example, the cohorts of patients selected for ambulatory care after ACDF may not be entirely the same as those selected for overnight hospital surveillance. To our knowledge, the Finnish Trial on Practices of Anterior Cervical Decompression and Fusion (FACADE), a prospective, parallel group, noninferiority randomized clinical trial (RCT), was the first level I study comparing ambulatory with inpatient care for any spinal surgery. The objective was to assess whether ambulatory care is noninferior to overnight hospital surveillance in functional outcome as measured by Neck Disability Index (NDI) in adults after ACDF for cervical radiculopathy.

## Methods

The FACADE trial was conducted in 3 tertiary neurosurgical centers in Finland (University Hospitals of Helsinki, Turku, and Oulu) from June 2019 to February 2021. The Helsinki and Uusimaa Hospital District Institutional Review Board approved the trial protocol (Supplement 1), which has been published.^15^ The study was conducted in accordance with the Declaration of Helsinki.^16^ All patients gave written informed consent. The reporting of this trial adhered to the Consolidated Standards of Reporting Trials (CONSORT) reporting guideline.^17^

### Participants

We enrolled patients between 18 and 62 years of age (the working-age range in Finland) who had clinical and imaging findings consistent with a diagnosis of cervical radiculopathy^18^ and symptoms severe enough to fulfill indications for surgery (NDI ≥30% on a scale from 0%-100%). We excluded individuals with cervical myelopathy, individuals who were unemployed, or individuals with comorbidities necessitating extended sick leave as well as individuals who had undergone a previous cervical operation. Detailed inclusion and exclusion criteria are shown in eTable 1 in Supplement 2.

### Surgical Procedure

A standard ACDF was performed as described in a previous study.^19^ All surgeries were performed during office hours by consultant neurosurgeons with at least 5 years of experience in cervical spine surgery. Interbody fusion was performed with a stand-alone cage (CeSPACE; Aesculap Inc). To ensure correct positioning of the cage, a confirmatory spinal radiograph was acquired at the end of the surgery.

### Randomization

**Figure 1.**
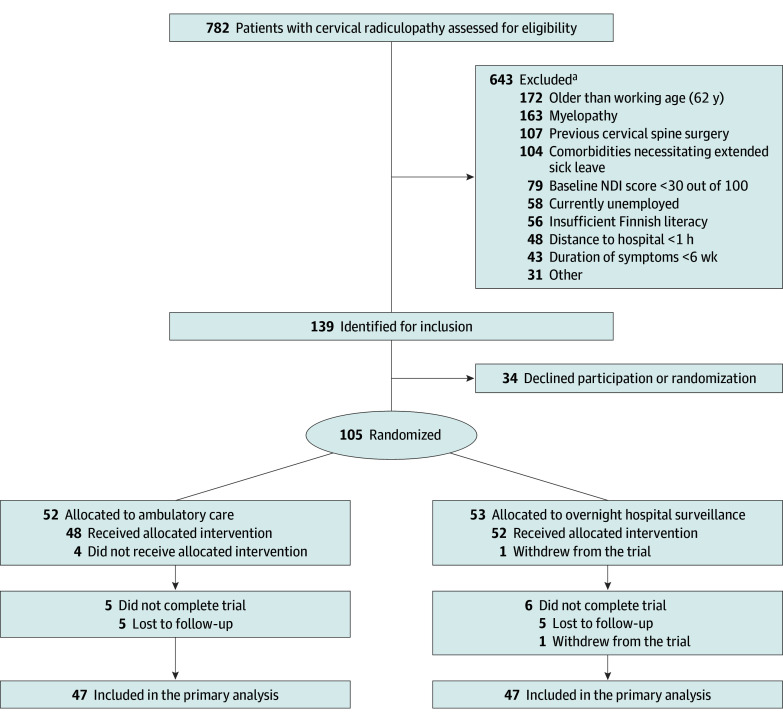
Diagram of Patient Flow in the FACADE Trial NDI indicates Neck Disability Index. ^a^Reasons for exclusion are provided in eTable 5 in Supplement 2, and information on missing data items is provided in eTable 10 in Supplement 2. Patients could have had more than 1 reason for exclusion. FACADE indicates Finnish Trial on Practices of Anterior Cervical Decompression and Fusion.

After surgery, we transferred patients to a recovery room for an approximately 3-hour follow-up. Once the patients regained full consciousness, a member of the trial personnel (anesthesiologist or neurosurgeon) ruled out immediate postoperative complications using a postoperative checklist. We then randomly assigned patients to either ambulatory care or overnight hospital surveillance (inpatient care) in a 1:1 ratio (Figure 1) using an electronic case report form system (eTable 2 in Supplement 2). Balanced random samples were generated through variable-size block randomization with the use of a computer randomization algorithm. After randomization, participants were transferred to the surgical ward.

### Blinding

Since blinding of patients was not possible, we interpreted the results of the trial according to a blinded data interpretation scheme,^20^ with minor refinements. Briefly, before the completion of the follow-up, the FACADE Writing Committee (eAppendix in Supplement 2) contemplated the possible interpretations of the data, agreed on predefined interpretative commitments, and issued a plan for an upcoming blinded data interpretation meeting. An independent statistician (T.C.) then carried out data analyses and chaired the subsequent blinded data interpretation meeting. In the meeting, the blinded results from the data analyses were presented to the Writing Committee, with treatment groups labeled as group A and group B. The Writing Committee considered the results in light of the predefined interpretative commitments and agreed in writing on the correct interpretation. Once the interpretation was chosen, the randomization code was broken, the interpretation was finalized, and the minutes were signed by all members of the Writing Committee.

### Interventions

After being transferred to a postoperative ward, patients were allowed to eat and ambulate freely. The use of a postoperative collar was left to the discretion of each participant. Postoperative pain was primarily managed using nonsteroidal anti-inflammatory drugs, acetaminophen, and weak opioids.

#### Ambulatory Care

In the ambulatory care group, patients were kept under surveillance for 6 to 8 hours after ACDF and then were discharged. Before being discharged, they were assessed by a study nurse using a specific checklist (eTable 3 in Supplement 2) to ensure there were no constraints for discharge.

#### Overnight Hospital Surveillance

In the overnight hospital surveillance group, patients were kept under surveillance in the hospital ward for 24 hours or longer. As with the ambulatory care group, the inpatient group was assessed for discharge eligibility before being discharged.

### Outcomes

The primary outcome was NDI at 6 months. The NDI is a validated patient-reported measure of neck pain–related disability. It consists of 10 questions with a 6-point response scale ranging from 0 (no disability) to 5 (full disability). The numerical response for each question is summed for a score of 0 to 50 points, which is converted to a percentage out of 100%; higher percentages indicate worse outcomes and more symptoms. We used a validated Finnish version of the NDI.^21^

Secondary outcomes included neck and arm pain, which were both self-measured using a numerical rating scale of 0 (no pain) to 10 (worst imaginable pain)^22^; return to previous daily activities and return to work, answered with a yes or no; Work Ability Score, self-assessed current work ability compared with lifetime best using an 11-point numerical rating scale with a possible score of 0 (completely unable to work) to 10 (work ability at its best)^23^; duration of sick leave in days; and EuroQol-5 Dimension-5 Levels [EQ-5D-5L] utility score, a health-related quality-of-life self-measure with a range of 0 (condition as bad as being dead) to 1 (full health).^24^ We used the Swedish value set and scoring algorithm^25^ to calculate the EQ-5D-5L utility scores because the Finnish scoring algorithm was not yet available.

All questionnaires were administered at baseline and at each follow-up time point (1 week, 2 weeks, 3 weeks, 1 month, 3 months, 6 months) (eTable 4 in Supplement 2). At baseline, we also collected data on demographic and clinical characteristics.

The patients’ general satisfaction was assessed at 6 months with the question “If you were to choose again, would you choose an operative treatment?” (response choice of yes or no). Operative success was assessed by patients subjectively at each follow-up time point using the modified Odom criteria,^26,27^ a rating scale ranging from poor to excellent. We considered the first and second categories (excellent and good) as a successful outcome and, conversely, the last 2 categories (fair and poor) as an unsuccessful outcome.

All outcomes were collected using an electronic case report form. At each follow-up time point, patients were automatically prompted to enter the required information into the system.

### Complications and Adverse Events

Patients were encouraged to contact the hospital in case of any adverse effects of ACDF. Additionally, at each follow-up, we queried the participants for potential adverse events and screened their medical reports for such events. We categorized adverse events as serious or minor. Serious adverse events were death, cardiovascular events, deep vein thrombosis, pulmonary embolism, systemic infection, postoperative neck hematoma, postoperative monoplegia or tetraplegia, or permanent dysphagia or dysphonia. Minor adverse events were local infection, periodic dysphagia, or dysphonia.

### Statistical Analysis

We set the noninferiority margin between ambulatory care and overnight hospital surveillance at a minimal important difference of 17.3 percentage points (NDI) to reflect the minimum clinically important difference suggested by Parker and colleagues.^28^ Thus, to achieve 90% power with a 2-sided type I error rate of 2.5%, assuming no difference between treatment groups, equal sample sizes, and a 15% dropout rate, the required sample size was 52 patients per group (total, 104).^15^

In the primary analysis, based on the intention-to-treat principle, we used a repeated-measures mixed model (RMMM) with the patient as a random factor (repeated measurements at 1, 3, and 6 months), the baseline value as a covariate, and assuming an independent variance-covariance structure and a generalized Satterthwaite approximation of *df* of a *t* distribution. As the RMMM allows for analysis of unbalanced datasets without imputation, we analyzed all available data. Noninferiority was claimed if the upper limit of the 2-sided 95% CI (CI based on difference in means in the NDI) was smaller than the minimal important difference in the primary comparison.^17^ NDI was the only outcome used to assess noninferiority.

To avoid falsely claiming noninferiority, we also conducted per-protocol and as-treated analyses. The per-protocol analysis included the overnight hospital surveillance group and patients in the ambulatory care group who adhered to the assigned treatment protocol. In the as-treated analysis, the groups were analyzed according to their last treatment modality (ambulatory care or overnight hospital surveillance).

We assessed all secondary outcomes with an equivalence hypothesis. However, since the trial was not necessarily powered for these comparisons and to avoid issues with multiplicity, we considered secondary outcome analyses to be exploratory or hypothesis generating.^17^ We used RMMM for all continuous outcomes (linear models) and binary outcomes (logistic models) when the outcome was registered at multiple time points after surgery. The only exception was the return-to-work outcome, for which standard logistic regression was used due to the outcome structure. For outcomes registered only at 6 months, a standard linear or logistic model was used and adjusted for baseline outcome values when available.

Data analyses were conducted in August 2022. Stata, version 15 (StataCorp LLC), was used.

## Results

### Patient Characteristics 

A total of 105 patients (mean [SD] age, 47.0 [7.9] years; 54 women [50%] and 51 men [50%]) were randomly assigned to ambulatory care (n = 52) or overnight hospital surveillance (n = 53). One patient in the overnight hospital surveillance group withdrew from the study immediately after randomization. The baseline characteristics of the 2 groups were similar (Table 1). The most frequently operated cervical spine levels in the ambulatory care group (70 levels operated) and overnight hospital surveillance group (61 levels operated) were C5-6 (38 [54%] and 22 [36%]) and C6-7 (29 [42%] and 36 [59%]). Additionally, 79 patients (75%) had a 1-level ACDF and 26 (25%) had a 2-level ACDF (eTable 6 in Supplement 2). After loss to follow-up (10 [10%]), there were 47 patients in the ambulatory care group and 47 patients in the overnight hospital surveillance group (Figure 1).

**Table 1.** Baseline Demographic and Clinical Characteristics of Patients^a^

Characteristic	Patients, No. (%)	
	Ambulatory care group (n = 52)	Overnight hospital surveillance group (n = 53)
Age at allocation, mean (SD), y	47 (7)	47 (9)
Sex		
Women	25 (48)	29 (55)
Men	27 (52)	24 (45)
Dominant hand affected	21 (40)	19 (36)
Work Ability Score, mean (SD)^b^	4.4 (2.2)	3.9 (2.4)
Patient’s perception of job demands: heavy	31 (60)	30 (59)
Ability to work normally regardless of the symptoms?	17 (33)	29 (56)
Duration of symptoms, median (IQR), d^c^	180 (100-300)	162 (90-365)
Duration of preoperative sick leave, median (IQR), d^c^	27 (0-90)	41 (0-92)
Prior treatments: physiotherapy	31 (60)	37 (70)
Patient’s use of pain medication		
NSAIDs	39 (75)	38 (72)
Opioids	23 (44)	18 (34)
Neuropathic pain medication	24 (46)	24 (45)
Neck Disability Index, mean (SD), %^d^	48 (12)	45 (10)
Neck pain at rest, mean (SD)^e^	5.8 (2.0)	5.2 (2.0)
Arm pain at rest, mean (SD)^e^	6.4 (1.9)	6.7 (1.7)
EQ-5D-5L utility score, mean (SD)^f^	0.76 (0.10)	0.75 (0.08)

Abbreviations: EQ-5D-5L, EuroQol-5 Dimension-5 Levels; NSAID, nonsteroidal anti-inflammatory drug.

^a^ Patients were included in groups as they were randomized.

^b^ Work Ability Score is an 11-point numerical rating scale for assessing current work ability compared with lifetime best, with a score range of 0 (completely unable to work) to 10 (work ability at its best).

^c^ Available for 101 of 105 patients.

^d^ Neck Disability Index is a validated, self-reported measure for neck pain and radiculopathy consisting of 10 questions that can be answered on a 6-point scale ranging from 0 (no disability) to 5 (full disability). The numerical response for each item is summed for a score of 0 to 50 and then converted to a percentage out of 100%, with higher percentages indicating worse outcomes and more symptoms.

^e^ Arm and neck pain were assessed with an 11-point numerical rating scale ranging from 0 (no pain) to 10 (worst imaginable pain).

^f^ EQ-5D-5L is a standardized measure of health-related quality of life that provides a utility score based on 5 dimensions ranging from 0 (condition as bad as being dead) to 1 (full health).

Of all patients assessed for trial eligibility, 643 were not included, mainly due to being older than 62 years (172 [27%]), having myelopathy (163 [25%]), and having undergone previous cervical spine surgery (107 [17%]). Other reasons for nonparticipation are given in eTable 5 in Supplement 2.

### Primary and Secondary Outcomes

**Figure 2.**
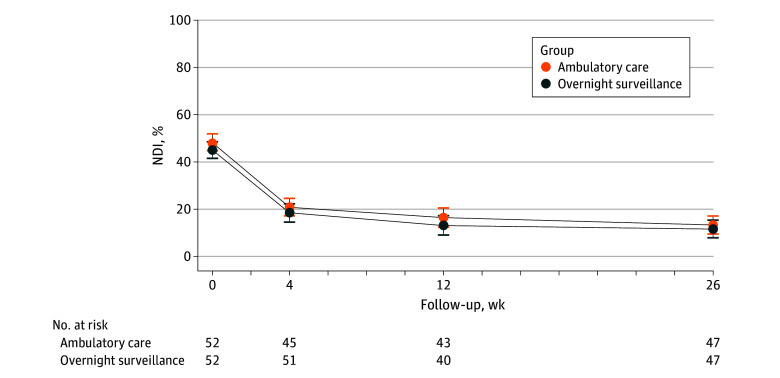
Neck Disability Index (NDI) Score Over Time The NDI is a validated, self-reported measure for neck pain and radiculopathy consisting of 10 questions that can be answered on a 6-point scale ranging from 0 (no disability) to 5 (full disability). The numerical response for each item is summed for a score of 0 to 50 and then converted to a percentage out of 100%, with higher percentages indicating worse outcomes and more symptoms. Error bars represent 95% CIs.

At the primary outcome assessment follow-up at 6 months, the mean NDI was 13.3% (95% CI, 9.3%-17.3%) in the ambulatory care group and 12.2% (95% CI, 8.2%-16.2%) in the overnight hospital surveillance group, with a between-group mean difference of 1.1 (95% CI, −4.6 to 6.8) percentage points (Table 2, Figure 2).

**Table 2.** Primary and Secondary Outcomes at 6-Month Follow-Up^a^

Outcome	Mean (95% CI)		Between-group mean difference (95% CI), percentage points	*P* value^b^
	Ambulatory care group (n = 47), %	Overnight hospital surveillance group (n = 47), %		
**Primary outcome**				
Neck Disability Index, %^c^	13.3 (9.3 to 17.3)	12.2 (8.2 to 16.2)	1.1 (−4.6 to 6.8)	.71
**Secondary outcomes**				
Arm pain^d^	1.7 (1.0 to 2.3)	1.6 (0.9 to 2.2)	0.1 (−0.8 to 1.0)	.82
Neck pain^d^	2.1 (1.5 to 2.8)	1.8 (1.2 to 2.5)	0.3 (−0.7 to 1.2)	.54
Return to previous activities, %^e^	82 (69 to 94)	92 (84 to 100)	−10 (−25 to 4)	.17
Return to work, %^f^	89 (79 to 99)	92 (84 to 100)	−3 (−16 to 10)	.66
Postoperative sick leave, mean (SD), d^g^	35 (40)	31 (26)	4.02 (−9.2 to 17.2)	.54
Work Ability Score^h^	7.5 (6.8 to 8.3)	7.8 (7.1 to 8.6)	−0.3 (−1.3 to 0.7)	.56
EQ-5D-5L utility score^i^	0.94 (0.91 to 0.96)	0.94 (0.92 to 0.96)	0.00 (−0.03 to 0.03)	.80
Patient satisfaction, %^j^	97 (83 to 99)	95 (81 to 97)	3 (−6 to 11)	.56
Operative success, %^k^	82 (69 to 94)	82 (69 to 94)	0 (−17 to 17)	>.99
Postoperative dysphonia^l^	0.7 (0.1 to 1.2)	0.5 (−0.0 to 1.0)	1.7 (−0.6 to 0.9)	.67
Postoperative dysphagia^l^	0.3 (−0.1 to 0.8)	0.1 (−0.3 to 0.5)	0.2 (−0.4 to 0.8)	.52

Abbreviation: EQ-5D-5L, EuroQol-5 Dimension-5 Levels.

^a^ Complete dataset of primary and secondary outcomes at different time points is provided in eTable 7 in Supplement 2.

^b^ Two-sided *P* < .05 indicated statistical significance.

^c^ Neck Disability Index is a validated, self-reported measure for neck pain and radiculopathy consisting of 10 questions that can be answered on a 6-point scale ranging from 0 (no disability) to 5 (full disability). The numerical response for each item is summed for a score of 0 to 50 and then converted to a percentage score out of 100%, with higher scores indicating worse outcomes and more symptoms.

^d^ Arm and neck pain were self-assessed with an 11-point numerical rating scale ranging from 0 (no pain) to 10 (worst imaginable pain).

^e^ Self-reported measure for returning to previous leisure activities, with yes or no as the answer choices.

^f^ Self-reported measure for returning to work, with yes or no as the answer choices.

^g^ Postoperative absence from work.

^h^ Work Ability Score is an 11-point numerical rating scale for assessing current work ability compared with lifetime best, with a score range of 0 (completely unable to work) to 10 (work ability at its best).

^i^ EQ-5D-5L is a standardized measure of health-related quality of life that provides a utility score based on 5 dimensions ranging from 0 (condition as bad as being dead) to 1 (full health).

^j^ Global satisfaction with the treatment was assessed with the question “If you were to choose again, would you choose an operative treatment?,” with yes or no as the answer choices.

^k^ Assessed with modified Odom criteria using a scale ranging from poor to excellent. Excellent and good ratings were considered to be a successful outcome of the operation.

^l^ Assessed with an 11-point numerical rating scale ranging from 0 (no dysphonia/dysphagia) to 10 (extreme dysphonia/odynophagia).

**Figure 3.**
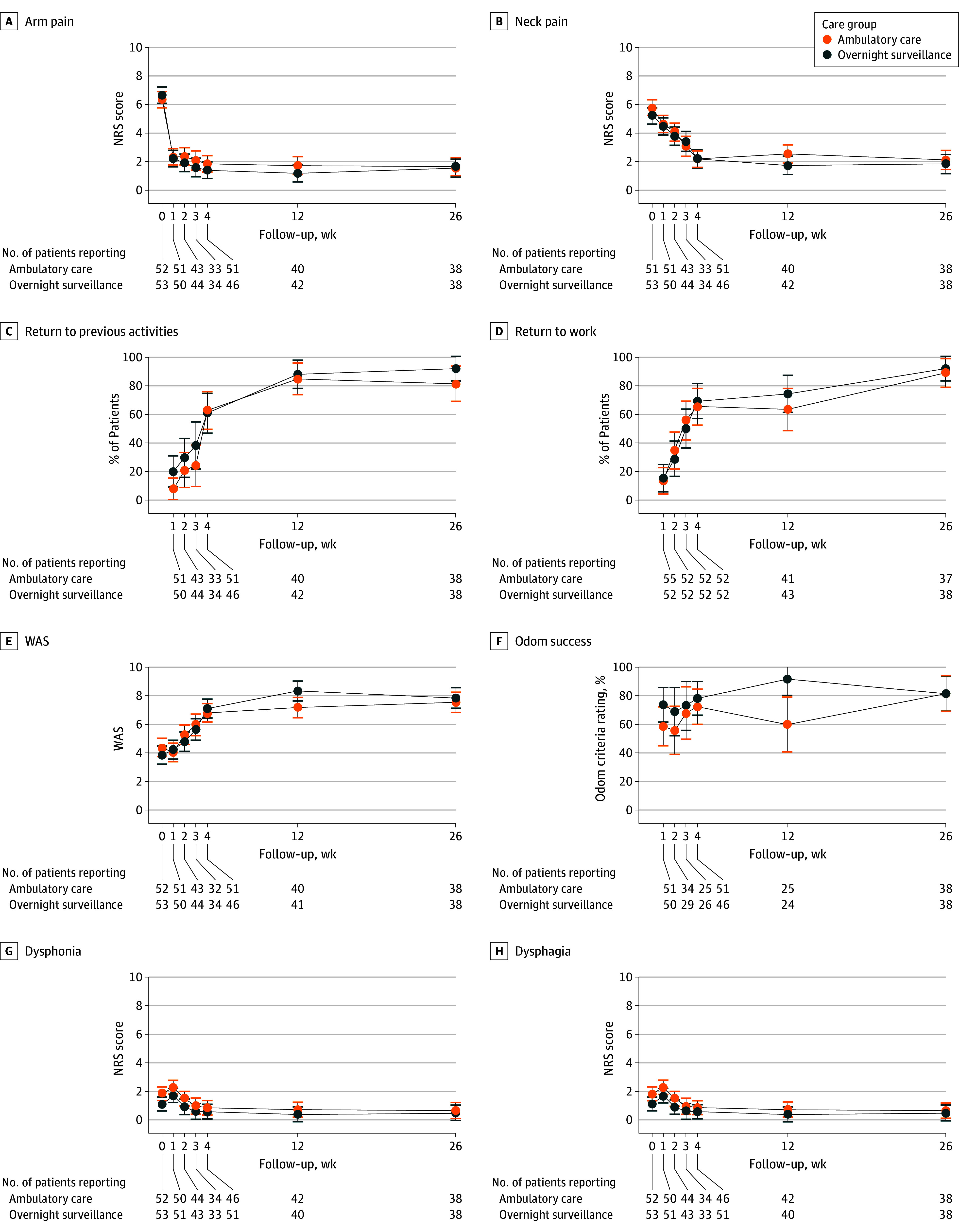
Trajectories of Secondary Outcomes in the Intention-to-Treat Analysis Error bars represent 95% CIs. NRS indicates numerical rating scale (score range varies for different measures); WAS, Work Ability Score (score range: 0 [completely unable to work] to 10 [work ability at its best]).

Data on all secondary outcomes are provided in Table 2, Figure 3, and eTable 7 in Supplement 2. We observed no significant between-group differences in any of the secondary outcomes at the 6-month follow-up. Apart from slightly lower Work Ability Score and subjective perception of operative success in the ambulatory care group at the 3-month follow-up (differences that were no longer evident at 6 months), there were no other significant between-group differences in the primary or any secondary outcomes at any of the assessment time points (1 week, 2 weeks, 3 weeks, 1 month, or 3 months).

### Crossovers, Acute Care Revisits, Rehospitalizations, and Adverse Events

Four patients (8%) crossed over from ambulatory care to overnight hospital surveillance because of postoperative neck pain and nausea (n = 2), excessive discharge (oozing) of the wound (n = 1), or transient urinary retention (n = 1). Seven patients (13%) in the ambulatory care group reported acute care revisits because of minor adverse events, including superficial wound infections (n = 3), transient dyspnea (n = 2), panic disorder (n = 1), and recurring cervical radiculopathy symptoms leading to reoperation 3 days after primary ACDF (n = 1). Of these patients, the latter 2 required rehospitalization. The only serious adverse event reported was a lower-extremity deep vein thrombosis diagnosed 4 days after the surgery in a patient in the overnight hospital surveillance group. Adverse events and crossovers are listed in eTable 8 in Supplement 2. Given the low number of crossovers in the ambulatory care group (n = 4), the findings of the per-protocol and as-treated analyses were almost identical to the intention-to-treat analyses (eTable 9 in Supplement 2).

## Discussion

The FACADE trial showed that ambulatory care resulted in noninferior functional outcomes compared with overnight hospital surveillance at 6 months after ACDF. The shift toward ambulatory care in spinal surgery may have considerable benefits, including patient comfort and reduced costs, assuming that outcomes and safety concerns are deemed noninferior. In the case of ACDF for cervical radiculopathy, the existing comparative observational evidence (derived from retrospective patient series or similar registry- or database-based studies) suggested equivalent outcomes without increased safety concerns.^3,4,5,6,11,12,13,14^

This trial contributes new, lower-bias evidence on outcomes for ACDF. By limiting participation to the working-age population in Finland (ages 18-62 years) as a reasonable proxy for lower-risk individuals eligible for ambulatory care, we were able to randomly assign patients to ambulatory care or overnight hospital surveillance. The main finding—that ambulatory care was noninferior to overnight hospital surveillance in functional outcome as measured by the NDI at 6 months after ACDF for this patient population—provides the first level I evidence comparing ambulatory with inpatient care for any spinal surgery.

Apart from efficacy, the question of safety warrants further consideration. For clinically relevant adverse events that are rare, even rigorous level I studies may not have the statistical power to fully address safety concerns. In the case of ACDF, the incidence of clinically relevant safety concerns after ACDF is low (0.1% to 9.1%).^3,4,5,6,11,12,13,14^ In the FACADE trial, the incidence of adverse events was similarly low (eTable 8 in Supplement 2), in line with the existing observational data.^3,4,5,6,11,12,13,14^ Although low incidence of clinically relevant safety concerns is an advantage, it also means that carrying out an RCT large enough to provide robust estimates on safety would be costly, onerous, and perhaps impractical. Certainly, the FACADE trial, although it provides rigorous evidence on functional outcomes, was underpowered to establish firm conclusions on the rate of rare adverse events, as almost any such trial would be.

The dilemma here comes into clear focus when considering the primary reason that many health care professionals still advocate overnight hospital surveillance after ACDF: the fear of neck hematoma, a potentially life-threatening consequence of this surgery. Although neck hematoma can be catastrophic for the affected patient, the risk is exceedingly low, with an estimated incidence of 0.1% to 0.3%.^3,4,5,13,14^ Within this small percentage of patients, there is limited evidence on how long after surgery a clinically relevant hematoma is likely to occur; however, studies suggest a likely timeframe of within 6 to 24 hours after surgery, with reported rates ranging from 65% to 100% during this surveillance period.^29,30,31^ At the same time, however, cases of hematomas occurring up to 6 days after surgery have also been documented, underscoring the unpredictable nature of this complication and the limitations inherent in surveillance. Ultimately, given the variability of neck hematoma and the low number of patients involved, it seems unlikely that estimates of the effect of ambulatory care vs hospital surveillance on such rare safety outcomes can ever be acquired with sufficient statistical power in an RCT. Therefore, the challenge remains regarding balancing the known benefits of ambulatory care for the majority of patients with managing the risk of severe but rare complications.

### Strengths and Limitations

To our knowledge, the FACADE trial provides the first low risk-of-bias evidence of the comparable efficacy of ambulatory vs inpatient care after ACDF. In a climate of vested interests both for and against ambulatory spinal surgeries, we consider the trial setting (publicly funded health care systems with no incentives to promote either of the studied treatment strategies) to be a strength of the study. We also consider the low number of individuals lost to follow-up (10% in the primary outcome) and the low incidence of crossovers (8%) as strengths.

This trial also had limitations. Our choice of the NDI as the primary outcome can be questioned. As a validated, patient-relevant, and disease-specific instrument developed to quantify the disability caused by radiating arm pain—the hallmark symptom of cervical radiculopathy and the primary reason most patients seek medical attention—the NDI was appropriate for our purpose. Additionally, the findings from the secondary outcomes, encompassing a broad range of symptoms and burden related to cervical radiculopathy, such as the patient perception of treatment success and their satisfaction with the treatment outcome, were all aligned with the findings of the NDI. While concerns about the validity of the chosen noninferiority margin are common in any noninferiority RCT, findings exclude any clinically significant difference between the 2 treatment strategies, regardless of the noninferiority margin.

Ambulatory spine surgery, including ACDF for cervical radiculopathy, is generally reserved for patients who are healthy, are undergoing less complex procedures, have adequate support systems, and live close enough to the surgical center to promptly address any postoperative complications. In line with these general guidelines, we restricted the sample to relatively healthy, employed individuals aged 18 to 62 years, resulting in a sample that constituted 18% of those assessed for trial eligibility.

Extrapolating the findings on the feasibility of ambulatory care to patients with a higher baseline risk must be carried out with caution. However, it is important to emphasize that the exclusion criteria were not based on risk aversion alone. Approximately half of the patients excluded from the trial were not disqualified due to high medical risk but rather due to criteria aimed at testing another hypothesis: less care could result in patients resuming daily activities and returning to work sooner. Some patients who were excluded for such criteria (eg, currently unemployed, needing extended sick leave due to comorbidities, or older than the Finnish working age limit of 62 years) might still be reasonable candidates for ambulatory care in other contexts. Future studies should explore the broader applicability of ambulatory care models to such individuals, as they may still benefit from early discharge protocols under appropriate conditions.

Robust evidence on benefits and downsides of medical interventions is essential for better decision-making and cost-benefit analyses. However, a judgment on the acceptable trade-off between benefits and downsides is inherently value laden, given the disparate priorities and risk tolerances among different individuals, organizations, and health care systems. For example, an advocate of ambulatory care is likely to consider the events leading to acute care revisits to be mostly unrelated to the treatment group allocation and the overall benefits (eg, >90% decrease in the need for inpatient care) to outweigh the crossovers from ambulatory care to overnight hospital surveillance (8%). Conversely, someone more skeptical about ambulatory care may well interpret the same data as suggesting that the downsides and increased risks of ambulatory care outweigh the benefits.

## Conclusions

Among patients deemed fit for early discharge, the results of the FACADE trial showed that ambulatory care was noninferior to overnight hospital surveillance in functional outcome with no increase in serious adverse events at 6 months after ACDF. Future studies should explore whether these findings apply to patients with a higher risk of adverse events, and clinicians and patients should consider the possibility of rare adverse events following this procedure before selecting ambulatory care.

## Notes

Supplement 1.
Trial Protocol

Supplement 2. **eAppendix.** The FACADE Investigators **eTable 1.** Inclusion and Exclusion Criteria Used in the FACADE Trial **eTable 2.** Interventions **eTable 3.** FACADE Discharge Checklist **eTable 4.** Schedule of Enrollment, Interventions and Assessments **eTable 5.** Reasons for Exclusion in 643 Patients **eTable 6.** Operated Levels and Number of Operated Levels **eTable 7.** Primary and Secondary Outcomes at Different Time Points **eTable 8.** Adverse Events, Re-admissions to Hospital and Crossovers (Number of Patients) **eTable 9.** Sensitivity Analyses: NDI Score in Per-Protocol and As-Treated Analyses **eTable 10.** Missing Data Items (No. of Patients)

Supplement 3.
Data Sharing Statement
