# Supplementary material for: Ambulatory Care vs Overnight Hospitalization After Anterior Surgery for Cervical Radiculopathy: The FACADE Randomized Clinical Trial
Source: JAMA Netw Open. 2024 Nov 27;7(11):e2447459. doi: 10.1001/jamanetworkopen.2024.47459 (PMC12124692; doi:10.1001/jamanetworkopen.2024.47459)
Supplement: Supplement 1. — Trial Protocol [file jamanetwopen-e2447459-s001.pdf]

## Trial Protocol, Statistical Analysis Plan and Blinded Data Interpretation

# Ambulatory Care versus Overnight Surveillance after Anterior Cervical Decompression and Fusion for Cervical Radioculopathy

### The FACADE Randomized Clinical Trial

Kimmo Lönnrot, MD, PhD; Simo Taimela, MD, PhD; Jarno Satopää, MD, PhD; Ilkka Saarenpää, MD, PhD; Ville Leinonen MD, PhD; Juri Kivelev, MD, PhD; Marja Silvasti-Lundell, MD, PhD; Johannes Förster, MD, PhD; Mikko Pitkänen, MD, PhD; Rahul Raj, MD, PhD; Mikko Kauppinen, MD; Riitta Westermarck, MD, PhD; Behnam Rezai-Jahromi, MD; Anniina Koski-Palkén, MD, PhD; Matti Seppälä, MD, PhD; Leena Kivipelto, MD, PhD; Jussi Antinheimo, MD, PhD; Miikka Korja, MD, PhD; Tomasz Czuba, BS, PhD; Teppo LN Järvinen, MD, PhD

## Table of Content

Trial protocol ........ 2

Statistical Analysis Plan and Blinded Data Interpretation Plan ........ 21

Minutes of the Statistical Analysis Plan and Blinded Data Interpretation ........ 38

## Trial protocol

# Finnish Trial on Practices of Anterior Cervical Decompression and Fusion (FACADE): A Protocol for a prospective randomised non-inferiority trial comparing Outpatient vs. Inpatient care

Kimmo Lönnrot[1,2], Simo Taimela[2], Pirjo Toivonen[2], Pasi Aronen[2], Anniina Koski-Palkén[1], Janek Frantzen[3], Ville Leinonen[4,5], Marja Silvasti-Lundell[6], Johannes Förster[7], and Teppo Järvinen[2], for the FACADE investigators.

Author affiliations

1. Department of Neurosurgery, University of Helsinki and Helsinki University Hospital, Helsinki, Finland
2. Finnish Centre for Evidence-Based Orthopedics (FICEBO), Department of Orthopedics and Traumatology, University of Helsinki and Helsinki University Hospital, Helsinki, Finland
3. Division of Clinical neurosciences, Department of Neurosurgery, Turku University Hospital and University of Turku, Turku, Finland
4. Department of Neurosurgery, Kuopio University Hospital and University of Eastern Finland, Kuopio, Finland
5. Unit of Clinical Neuroscience, Neurosurgery, University of Oulu and Medical Research Center, Oulu University Hospital, Oulu, Finland
6. Division of Anaesthesiology, Department of Anaesthesiology, Intensive Care and Pain Medicine, University of Helsinki and Helsinki University Hospital, Helsinki, Finland
7. Department of Anaesthesia, Orthopaedic Hospital Orton, Helsinki, Finland

**Correspondence to**

Dr. Kimmo Lönnrot, Department of Neurosurgery, Helsinki University Hospital, Töölö hospital, Topeliuksenkatu 5, Helsinki 00260, Finland, tel+358-50-4270270, kimmo.lonnrot@hus.fi

**Keywords:** Anterior cervical decompression and fusion, cervical spine surgery, outpatient care, randomised controlled trial, protocol

**Word count (excluding title page, abstract, references, figures and tables):** 4,388

## ABSTRACT

**Introduction:** Although a great majority of Cervical radiculopathy syndrome (CRS) patients can successfully be treated nonoperatively, a considerable proportion experience persistent symptoms, severe enough to require neurosurgical intervention. During the past decade, cervical spine procedures have increasingly been performed on an outpatient basis and retrospective database analyses have shown this to be feasible and safe. However, there are no randomised controlled studies comparing outpatient care with inpatient care, particularly with emphasis on the patients' perception of symptom relief and their ability to return to normal daily activities and work.

**Methods and Analysis:** This is a prospective, randomised, controlled, parallel group non-inferiority trial comparing the traditional hospital surveillance (inpatient, patients staying in the hospital for 1-3 nights after surgery) to outpatient care (discharge on the day of the surgery, usually within 6-8 hours after procedure) in patients who have undergone anterior cervical decompression and fusion (ACDF) procedure. To determine whether early discharge (outpatient care) is noninferior to inpatient care, we will randomise 104 patients to these two groups and follow them for 6 months using the Neck Disability Index (NDI) as the primary outcome. We expect that early discharge is not significantly worse than the current care in terms of change in NDI. Noninferiority will be declared if the mean improvement for outpatient care is no worse than the mean improvement for inpatient care, by a margin of 17.3%. We hypothesize that a shorter hospital stay results in more rapid return to normal daily activities, shorter duration of sick leave and decreased secondary costs to health care system. Secondary outcomes in our study are arm pain and neck pain using the Numeric Rating Scale, operative success (Odom's criteria), patient's satisfaction to treatment, general quality of life (EQ-5D-5L), work ability score (WAS), sickness absence days, return to previous leisure activities, and complications.

E**thics and Dissemination:** The study was approved by the institutional review board of the Helsinki and Uusimaa Hospital District and duly registered at ClinicalTrials.gov. We will disseminate the findings of this study through peer-reviewed publications and conference presentations.



**Trial registration number:** ClinicalTrials.gov NCT03979443 (first registered June 7, 2019).

**Strengths of this study**

- Efficacy design: Strict eligibility criteria
- Special emphasis on the patients' perceptions of early recovery and their self-perceived ability to return to normal daily activities and work.
- Patient-partnership concerning the design of the study, the informational material, and the burden of the trial from the patient's perspective.

**Limitations of this study**

- Strict eligibility criteria possibly limit the generalizability of the findings.
- Participants awareness of the group assignment (lack of blinding) increases the risk of performance bias.

## INTRODUCTION

Cervical radiculopathy syndrome (CRS) or radiating arm pain caused by cervical nerve root irritation is a common complaint in otherwise healthy working-aged population with an annual incidence of approximately 0.8 per 1000 inhabitants (1). Conservative treatment is a viable alternative for the majority of CRS patients, as their symptoms are periodic and improve with time. However, a considerable proportion of CRS patients experience persisting symptoms severe enough to justify neurosurgical intervention. Anterior cervical decompression and fusion operation (ACDF), first described in 1950s by Cloward (2) and Smith-Robinson (3), has become the gold standard surgery for CRS (4,5). Originally, the ACDF procedure comprised of decompressing the nerve root followed by replacing the intervertebral disc by an autologous bone graft harvested from iliac crest to fuse the two adjacent vertebral bodies together. After the introduction of PEEK (Poly-Ethane-Ethene-Ketone) and titanium cages, autologous bone grafts are rarely used anymore, to avoid morbidity related to the donor site. With the advent of minimally invasive surgical techniques, anterior cervical fusions can now be achieved with reduced tissue trauma, pain and blood loss.

In most countries, the contemporary postoperative clinical practice after ADCF is to keep the patients under surveillance overnight for immediate postoperative complications, typically cervical swelling, neck hematoma and postoperative bleeding in the epidural space. However, a number of recent retrospective database analyses suggest that successful outpatient anterior cervical fusions can be done without an increased risk of complications (6–8). The surge of outpatient spine surgery, particularly in the United States, is also evident in the recent US-based clinical practice guidelines that advocate early discharge over inpatient care (9).

To date, no randomised trial has compared outpatient to inpatient care in patients who have undergone ACDF, particularly with a special emphasis on the patients' immediate perceptions of the care given and their self-perceived ability to return to normal daily activities and work. We will conduct a pragmatic, randomised, non-inferiority trial comparing the traditional hospital surveillance (inpatient) to outpatient care (same day discharge, within 6-8 hours after procedure) for patients undergoing ACDF procedure. Noninferiority of the new treatment (outpatient care) with respect to the reference treatment (inpatient care) is of interest on the premise that the new treatment has some other advantages, such as greater availability, faster recovery, reduced cost, less disabling, fewer adverse events (harms), or greater ease of administration (10). We hypothesize that with the current technique of performing ACDF, the outcome of patients in the outpatient care group is not worse than in the conventional postoperative strategy (inpatient care), exceeding the non-inferiority margin and with no significantly increased risk of harms. We further hypothesize that patients treated on an outpatient basis would perceive themselves to be less disabled, subsequently encouraging them to return more quickly to their normal daily activities and work. This will be assessed as our secondary objective.

## MATERIALS AND METHODS

### Overview of study design

We will start the FACADE as a single center prospective randomised non-inferiority study with the primary objective to compare the traditional strategy of keeping patients under hospital surveillance overnight (inpatient group) to a strategy of early discharge (6-8 hours after procedure, outpatient group) in patients having undergone ACDF procedure. We are planning to expand the FACADE to two or three other University Hospital centers in Finland if our recruitment is too slow. We hypothesize that ACDF performed with current techniques will have comparable outcomes and no increased harms in the early discharge arm vs. the conventional postoperative arm. Additionally, we hypothesize that patients treated on outpatient basis would perceive themselves as less disabled, subsequently encouraging them to return more quickly to their normal daily activities and work. This will be assessed as the secondary outcome.

### Ethical approval

The trial protocol was approved by ethical review at the institutional review board of the Helsinki and Uusimaa Hospital District on June 6, 2019 (1540/2019).

### Participant selection and recruiting process

We will screen all patients suffering from radiating arm pain referred to the department of neurosurgery at Helsinki University hospital for trial eligibility by the FACADE investigators. We will carry a standard clinical examination and MRI examination of the cervical spine. Patients with clinical and imaging findings consistent with a diagnosis of CRS and willing to undergo ACDF operation after being fully informed of the trial protocol and the benefits and potential harms of the surgery, will be evaluated for eligibility.

### Inclusion criteria

1) Cervical radiculopathy syndrome (CRS) unresponsive to non-operative treatment for at least six weeks or with severe progressive signs and symptoms of nerve root compression during conservative treatment of shorter duration.
2) CRS is defined as pain, paresis or paresthesia in corresponding nerve root distribution areas of C5, C6, C7 or C8.
3) Nerve root stenosis determined by magnetic resonance imaging at treatment level correlating to CRS/symptoms
4) Neck Disability Index score ≥30 out of 100
5) Age between 18 to 62 years
6) No previous cervical operations

7) Currently employed
8) No co-morbidities causing a need for a sick leave
9) Provision of informed consent from the participant
10) No contraindication for randomisation in postoperative check (see below)

**Exclusion criteria**

1) MRI finding inconsistent with patient's symptoms
2) Diagnosed osteoporosis or permanent use of oral corticosteroids
3) ACDF operation requiring plate or cage fixation with screws
4) Active malignancy
5) American Society of Anesthesiologists Physical Status Classification system (ASA) 4 and 5 patients (seriously ill patients)
6) Pregnancy
7) Abundant use of alcohol, drugs or narcotics
8) No possibility to be accompanied by an adult person over the first postoperative night after the surgery
9) Insufficient Finnish language skills
10) Distance to the closest hospital emergency more than 60 min

**Informed consent**

At the first appointment, we will provide the patients with detailed written and oral information of the trial and ask patients to sign a consent form. We will ensure that patients understand that the surgical procedure and the ensuing follow-up will be identical irrespective of study group allocation, and that the randomisation only occurs after the surgery. We will also inform the participants that participation in the study is entirely voluntary and any decision they make will not influence any future care. Participants will also be informed of their right to withdraw from the trial whenever they desire without the need to supply any reason for such decision, and in such cases their data acquired prior to withdrawal will be maintained in the study database and included in analysis to avoid bias. Patients who are eligible for the trial, but are not willing to undergo randomisation, will be asked to be included in a simultaneous, pragmatic follow-up cohort.

**Baseline assessment**

Our baseline assessment includes documentation of the following characteristics: gender, birth date, education, current employment status, hand dominance, time from the onset of symptoms, and recreational habits. We will also ask the participants to assess their physical workload (physically hard/demanding or not), their general health, and usage of pain medication. Finally, we will also document any prior conservative treatment (Table 1).

| Table 1: Baseline characteristics | OutP | InP |
|---|---|---|
| Age (years), mean (SD) | | |
| Gender (female/male), n (%) | | |
| Dominant hand affected, n (%) | | |
| Work Ability Score (WAS) | | |
| Physically demanding job, n (%) | | |
| Ability to work normally irrespective of the symptoms? n (%) | | |
| Participation in leisure time activities irrespective of the symptoms? n (%) | | |
| Duration of symptoms (days), mean (SD) | | |
| Sick leave duration (days) | | |
| Prior treatments (Physiotherapy) n (%) | | |
| NSAID Pain medication, n (%) | | |
| Opioid pain medication, n (%) | | |
| Neuropathic pain medication, n (%) | | |
| EQ-5D-5L, mean (SD) | | |
| Neck Disability Index (NDI) scores (0-100 NDI scale), mean (SD) | | |
| Neck pain at rest (0-10 NRS scale), mean (SD) | | |
| Arm pain at rest (0-10 NRS scale), mean (SD) | | |

**ACDF operation and anaesthesia**

A standard anesthetic procedure will be utilized. Every patient will be given preoperatively 10 mg diazepam, 1000 mg paracetamol, and 90 mg etoricoxib. During the operation, anaesthesia is maintained with propofol infusion with standard amount of fentanyl and rocuron.

FACADE neurosurgeons will carry out a standard ACDF operation as described previously (4). All FACADE neurosurgeons are members of the Finnish Neurosurgeons Association and have successfully completed at least 100 ACDF operations over the past five-year period. Interbody fusion is performed with a Polyetheretherketone (PEEK) cage (Cespage, Easculap, BBrauns, Germany) and a confirmatory x-ray will be acquired at the end of the operation for ensuring the correct positioning of the cage. We will instruct the patients not to wear any type of collar postoperatively.

**Randomisation and concealment**

After the surgery, we will take all patients to the recovery room for 2 to 3 hours for an immediate postoperative observation. When we have confirmed that the patients are fully conscious and co-operative, immediate postoperative complications are ruled out using a postoperative checklist and patients' final eligibility is confirmed. A member of the FACADE study group then carries out randomisation. The randomisation is a built-in property in the online electronic case report form (eCRF) system used in the trial. To minimise the risk of predicting the treatment assignment of the next eligible patient (to ensure concealment), we will perform randomisation with variable block size

(block size known only to the statistician with no involvement in the clinical care of the participants in the trial).

## Intervention

*Outpatient group (OutP)*

A ward nurse will evaluate all patients allocated to the Outpatient group approximately 6-8 hours after surgery using a standardised FACADE discharge checklist. If the patient fulfils all discharge criteria, he/she will be instructed on how to deal with any concerns and will be discharged. At discharge, we will document the time elapsed from operation and provide the patients with prescriptions to manage postoperative pain and an absence from work medical certificate for the first postoperative week.

*Inpatient group (InP)*

Patients allocated to inpatient care will be kept overnight. A neurosurgeon on duty will assess the "readiness" of the patient to be discharged using the FACADE discharge checklist. Identically to OutP group, at discharge we will document the time elapsed from operation and provide the patients with prescriptions for postoperative pain management and an absence from work medical certificate for the first postoperative week.

## Compliance to treatment allocation and possible cross over

If a patient allocated to OutP group does not consider herself/himself fit enough to be discharged or will not pass the discharge checklist (for example, due to severe nausea, neck swelling indicating a possible postoperative haematoma, insufficient relief of radiating upper extremity pain, severe neck pain, severe difficulties to swallow, a new paraesthesia or paresis on upper extremity, or a new postoperative dysphonia), we will keep her/him at the ward for as long as deemed necessary. We will consider these patients crossovers.

## Primary outcome measure

*NDI, Neck Disability Index*

Our primary outcome measure is the Neck Disability Index (NDI; scale 0-100, with higher scores indicating worse outcomes and more symptoms), a validated, neck-specific, patient reported measure of pain-related dysfunction (11,12) We will use a validated Finnish version of the NDI (13). The primary assessment time point is six months. We will also query the NDI at one and three months postoperatively, but this data is only intended to illustrate the trajectory of the treatment

responses (Table 2). We will consider 17.3% improvement from the baseline value as the minimal important difference (MID) for NDI (14).

**Secondary outcome measures**

*Neck and arm pain*

We will ask the patients to estimate the intensity of their neck pain (NRS-NP) and arm pain (NRS-AP) using a standard internet based Numeric Rating scale (eNRS) (15) at baseline, and at one week, one month and six months after the surgery (Table 2). We will use a 11-unit NRS scale ranging from 0 (no pain) to 10 (extreme pain). We will consider 2 points as the minimal important difference (MID) (15).

*Return to previous leisure activities*

Before the operation, each patient will be asked to name the most important daily leisure activity they are not able to perform because of the disease. At each follow-up time point (Table 2), participants will be asked to respond to the following question: “Have you been able to return to your leisure activity?” (“yes” or “no”)

*The single item work ability score (WAS)*

Patients will assess their ability to work according to single item work ability score (16) preoperatively/at baseline, at discharge after surgery, and 1 week, 1 month and 6 months after the surgery. The single-item WAS is an 11-point NRS in which patient will assess his or her current work ability compared with the life-time best, with a possible score of 0 (“completely unable to work”) to 10 (“work ability at its best”).

*The duration of sick leave*

We will record the duration of sick leave, i.e., the number of sickness absence days both before and after the operation. The number of sickness absence days will be treated as a continuous variable.

**Other (ancillary) outcome measures**

*EQ-5D-5L*

EQ-5D-5L is a standardized health-related quality of life instrument for assessing a patient’s general health and treatment outcome (17) and details a patient’s self-assessed health profile. The scores can be converted into quality-adjusted life years (QALYs). An improvement of 0.24 QALY will be considered clinically relevant (14).

*Patient satisfaction*

We will elicit patients' global assessment of satisfaction to the treatment at six months after operation with this question: "If you were to choose again, would you choose an operative treatment?" ("yes" or "no").

*Operative success*

Operative success will be assessed at each follow-up time point (Table 2) by the modified Odom's criteria (18), in which patient subjectively rates the perception of operative success from poor to excellent (19). We will consider the first and second categories ('excellent' and 'good') as a successful outcome of the operation and conversely, last two categories ('fair' and 'poor') as an unsuccessful outcome.

*Complications and adverse effects*

Complications directly related to the interventions will be registered. Postoperative dysphonia and odynophagia will be assessed with 0 to 10 numerical rating scales NRS-DP (NRS-dysphonia) and NRS-OP (NRS-odynophagia) at each postoperative time point (20,21). NRS will be assessed on an 11-unit scale ranging from 0 (no dysphonia/odynophagia) to 10 (extreme dysphonia/odynophagia).

The participants will also be encouraged to contact the hospital if any adverse effects occur. The patient's contact with the health care system will be registered at every follow-up visit. Potential adverse effects (AE) will be categorized as serious adverse effects (SAE) or minor adverse effects (MAE). Death, cardiovascular events, deep venous thrombosis, pulmonary embolism, systemic infection, postoperative neck haematoma, postoperative monoplegia or tetraplegia, permanent dysphagia or dysphonia will be categorised as SAEs, and local infection, periodic dysphagia or dysphonia will be categorised as MAEs. The number and severity of complications and adverse effects will be assessed.

**Follow-up**

The full follow-up process is outlined in table 2. Our primary method for collecting follow-up information is an electronic questionnaire. A link to this questionnaire is sent via email to all patients at all follow-up time points. To assess the participants' ability to return to everyday activities and return to work, we will also contact the participants by phone one week after surgery. Special attention will be paid to patient's self-perception of ability to return to work. If a patient is able to return to work, weekly follow-up will be terminated. If a patient does not feel able to return to work, the sick leave will be extended and a phone contact is scheduled on the next week. Weekly contacts will be continued for up to three weeks, if needed. In case of further need for sick leave beyond one month, a patient will be invited to an outpatient follow-up visit. We collect data on healthcare resource

utilization at the 1-, 3-, and 6-month follow-ups. In addition, we also encourage the patients to contact the FACADE study nurse if they encounter any problems that require medical attention at any time over the course of the follow-up.

**Table 2:** The follow-up assessments and data collection timetable.

| Assessment | BL | SG | 1 w | 2w | 3w | 1mo | 3mo | 6mo |
|---|---|---|---|---|---|---|---|---|
| Informed consent | X | | | | | | | |
| Baseline form | X | | | | | | | |
| MRI | X | | | | | | | (X) |
| Randomisation | | X | | | | | | |
| Adverse effects | | X | X | (X) | (X) | X | X | X |
| WAS | X | | X | (X) | (X) | X | X | X |
| Return to work | | | X | (X) | (X) | X | X | X |
| Return to previous activities | | | X | (X) | (X) | X | X | X |
| ODOM | | | X | (X) | (X) | X | X | X |
| Patients satisfaction to the treatment | | | | | | | | X |
| Health resource utilization | | | X | (X) | (X) | X | X | X |
| Clinical examination | X | | | | | (X) | | (X) |
| NDI | X | | | | | X | X | X |
| NRS-AP | X | | X | (X) | (X) | X | X | X |
| NRS-NP | X | | X | (X) | (X) | X | X | X |
| EQ-5D-5L | X | | | | | | | X |

Abbreviations:
BL: baseline; SG: surgery; 1 wk: 1 week postoperatively; 1mo: 1 month postoperatively; WAS: Work Ability Score; ODOM: Operative success; NDI: Neck Disability Index; NRS-AP: Numeric Rating Scale Arm Pain; NRS-NP: Numeric Rating Scale Neck Pain.
() if required

### Adherence and loss to follow-up

To safeguard against potential loss to follow-up, we will: Exclude individuals likely to pose suboptimal adherence to study follow-up, obtain verified contact information from each consented participant, and remind the participants of upcoming follow-up visits. All attempts will be made to make follow-up as convenient as possible for the patients. Participants are not required to visit the outpatient clinic postoperatively. Only in case of suboptimal operative result or any other possible concern related to care, a patient will be offered an opportunity to be assessed at the outpatient clinic. Otherwise, follow-ups will be carried out using phone interviews and/or electronic questionnaires to be filled online. The follow-up schedule will not incur any costs to the participants. We will monitor the adherence to follow-up throughout the trial and send reminders to patients who fail to return follow-up questionnaires.

### Missing items

We will use multiple imputation by chained equations to handle missing data for those statistical analyses that cannot handle occasional missing values (22). All variables to be included in the final

analyses will be forced in the chained equations imputation model and possibly including auxiliary variables available in the data set. The imputation algorithm, called fully conditional specification (FCS), uses specific univariate model for each variable and, for each specific imputed dataset, iteratively imputes each variable with missing values and uses the imputed values in the imputation of other variables.

**Sample size**

The trial is primarily designed to ascertain whether outpatient care is non-inferior to inpatient care, at 6 months after surgery, with NDI as the primary outcome. Only one primary analysis will be used to assess non-inferiority. The trial is powered to detect a minimal important difference (MID) in the NDI score between the two study groups. We set the MID for NDI (17.3%) as our margin of noninferiority Δ based on results by Parker et al. (14). At the 6-month time point, noninferiority can be claimed if the lower limit of the CI [based on difference in means in the NDI] is greater than the MID in the primary comparison. According to the CONSORT-statement for noninferiority and equivalence trials (10), secondary outcomes can be managed using either a superiority or equivalence framework. In our trial, all secondary outcomes will be assessed with an equivalence hypothesis, but since our trial is not necessarily powered for these comparisons, and to avoid issues with multiplicity, we consider them exploratory or hypothesis-generating.

The sample size calculation is based on the primary outcome measure, NDI at 6 months post-surgery. The sample size is approximated using equation (Equation 3.12 in Chow: Sample Size Calculations in Clinical Research, Third Edition CRC Press 2018) for non-inferiority test:

$$n_1 = \varkappa n_2 \ and \ n_2 = \frac{\left(z_\alpha + z_\beta\right)^2 \sigma^2 (1 + {}^1\!/_{\varkappa})}{(\varepsilon - M)^2}$$

Assuming no difference between treatment arms ($\varepsilon = 0$ in NDI score improvements, equal sample sizes ($\varkappa = 1$), the standard deviation 23%, a margin of noninferiority Δ of 17.3 %, one-sided 2.5% statistical significance criteria ($z_\alpha$= 1.96), and 90% statistical power ($z_\beta$=1,28) , we will need 44 patients per study group. Assuming a drop-out rate 15%, the group size increases to 52 patients. Accordingly, we set the recruitment target at 104 patients.

**Statistical analysis**

We will follow primarily intention-to-treat (ITT) principle in all our analyses. In the ITT analyses, the participants are included as randomised. Per-protocol and on-treatment analyses will also be used to avoid falsely claiming non-inferiority. Summary statistics will be given as mean (with SD) for

continuous variables and as frequencies (with %) for categorical variables. Repeated measures mixed model (RMMM) analysis will be used for all continuous variables (both primary and secondary outcomes) where regression coefficients are allowed to differ between study subjects. Statistical significance is set to two-sided 5% level. The RMMM analysis allows the use of all available observations in the data set, so the full data set (data set without multiple imputation) will be used in the analysis. Logistic regression will be used to assess categorical variables. R statistical software (R Core Team (2013). R: A language and environment for statistical computing. R Foundation for Statistical Computing, Vienna, Austria. URL http://www.R-project.org/.) will be used for analyses.

The number and proportion of individuals eligible for and compliant with each follow-up will be documented. We will also carry out an analysis of the demographic and prognostic characteristics between the individuals who withdrew and those who remained in the study.

**Safety considerations**

To safeguard against possible complications related to postoperative hematoma, all patients are required to live within 60 min distance from a surgical emergency unit.

**Data management and sharing**

All study data will be stored in an electronic case report form (eCRF). Upon receipt of the data, the FACADE personnel, blinded to the group allocation, will make a visual check of the data and query all missing, implausible, and inconsistent data. Hospital patient records will also be used to collect missing data and in interpreting inconsistent or implausible data. Participant files will be maintained in storage (both in electronic and paper format) at the coordinating centre for a period of 15 years after completion of the study.

Data generated by our research will be made available as soon as possible and will be available upon reasonable request. Data access request will be reviewed by FACADE steering group. Requestors will be required to sign a Data Access Agreement.

**Blinded data interpretation**

As previously (23,24), we will interpret the results of the trial according to a blinded data interpretation scheme (25). In brief, an independent statistician will provide the Writing committee of the FACADE trial with blinded results from the analyses with the groups labelled group A and group B. The Writing Committee will then interpret the results until a consensus is reached and agree in writing on all alternative interpretations of the findings. Once a consensus is reached, we will record the minutes of this meeting in a document coined “statement of interpretation,” which is signed by all members of the Writing Committee. Only after reaching this common agreement, the data manager and the independent statistician will break the randomisation code and the correct interpretation is chosen.

A manuscript will then be prepared and finalized for the publication of the results. Detailed minutes of blinded data interpretation meetings will be provided as a supplement to the trial manuscript.

**Patient and Public Involvement**

To achieve more patient-friendly design for our trial (26), we recruited six patient experts from the European Patients' Academy on Therapeutic Innovation (EUPATI Finland, https://fi.eupati.eu/). They were asked to review the consent form and the study questionnaires and to pilot the online eCRF before these were submitted to Ethical institutional review board. Among piloting online eCRF, these experts were asked to assess the burden of the intervention and time required to participate in the research, which both they estimated to be reasonable. After the FACADE-study is completed we will contemplate together with EUPATI Finland how to share the study results to the public.

**Data safety and monitoring committee**

The purpose of the Data Safety and Monitoring Committee (DSMC) is to advise the FACADE Investigators regarding the continuing safety of the trial participants. The DSMC is comprised of two clinical experts (neurosurgeons) with prior trial experience and a clinical trial methodologist. All members are independent of the trial investigators and have neither financial nor scientific conflicts of interest with the trial.

**Ethical considerations and dissemination**

All patients included in FACADE will sign an Ethics Board-approved consent form that describes this study and provides sufficient information for patients to make an informed decision about their participation. All participating centers must obtain Ethics Board approval from their institution for the study protocol, the consent form template, the CRFs, and any additional protocol amendments. Any protocol amendments will be communicated to the site investigators, the Ethics Board, trial participants, and trial registries as necessary.

Information about study patients will be kept confidential and will be managed in accordance with the following rules: 1) All study-related information is stored securely at the clinical site, 2) All possible study patient information in paper form is stored in locked file cabinets and is accessible only to study personnel, 3) All CRFs are identified only by a coded patient number, 4) All records that contain patient names, or other identifying information, are stored separately from the study records that are identified only by the coded patient number, and 5) All local databases are password protected.

## DISCUSSION

The rationale for the FACADE trial includes: 1) the growth in popularity of ACDF operations carried out as outpatient procedures over the last decade; 2) a lack of rigorous (RCT) evidence verifying the efficacy and safety of outpatient ACDF surgery; 3) uncertainty regarding the possible effect of hospitalization (vs. early discharge) on the recovery of patients to previous leisure activity and return to work.

According to CONSORT statement for noninferiority trials (27), a noninferiority trial seeks to determine whether a new treatment is not worse than a reference treatment by more than an acceptable amount. Because proof of exact equivalence is impossible, a pre-stated margin of noninferiority (Δ) for the treatment effect in a primary patient outcome is defined. Noninferiority of the new treatment with respect to the reference treatment is of interest on the premise that the new treatment has some other advantage, such as greater availability, reduced cost, less invasiveness, fewer adverse effects (harms), or greater ease of administration. In this FACADE trial, we primarily set out to determine whether the outcome of outpatient group is non-inferior to current standards of care (overnight stay) at 6 months postoperatively. Given that the hallmark symptom of this disease – and also the primary reason the patients seek for medical attention – is the disability caused by radiating arm pain, we felt that the Neck Disability Index is the most appropriate primary outcome for our trial. Although one can argue that rapid return to normal daily activities and work would be a better indication that a person has reached a stable health status, such contention may not be entirely accurate. Comprehensive data on sustainable return to work (RTW) shows that a variety of personal and social factors have positive and negative influences on sustainable RTW (28). Obviously, the social environment and how it interrelates with personal factors like attitudes towards work and self-efficacy play a role alongside the alleviation of neck pain as predictors of RTW.

The appropriateness of the chosen margin of noninferiority Δ is obviously a critical methodological issue regarding the validity of any non-inferiority trial. The CONSORT guidance states that it should be specified and preferably justified on clinical grounds, given that a too large margin of noninferiority Δ will increase the risk of accepting a truly inferior treatment as noninferior (27). Given this, it could be argued that we should have picked a value smaller than the MID of the NDI as our margin of noninferiority Δ, but as the MID defines the *minimal* important difference, we consider this decision justified.

Although the safety and particularly the long-term – preferably sustained – success is of indisputable primary importance in any surgery, early discharge might pose advantages that currently remain unaddressed. In essence, while keeping patients hospitalized overnight after surgery might increase

immediate postoperative safety, it may come at the risk of overcautiousness, increased perceived disability, and increased morbidity/disease toll. Given the existing evidence – primarily from registry-based studies – that suggests that ACDF surgery can be successfully performed as an outpatient procedure without increased safety concerns, we felt intrigued to test a hypothesis that early discharge encourages patients to return to their daily activities and work earlier than if they are hospitalized overnight. This hypothesis is the other primary study question of our prospective randomised trial.

**Author contributions**

KL, TLNJ, and ST designed the trial. KL, TLNJ, ST, PT, PA, AK-P, JF, MS-L, JF and VL have been involved in drafting the manuscript or revising it critically for important intellectual content. All authors read and approved the final manuscript.

*Writing Committee:* KL, ST, PT, PA, AK-P, JF, VL, MS-L, JF and TLNJ.

*Steering Committee:* Kimmo Lönnrot (Chair), Simo Taimela (Co-Chair), Teppo Järvinen (Co-Chair), Ville
Leinonen, Janek Frantzen, Pirjo Toivonen (Research Coordination).

*Methods Centre (Helsinki University Hospital):* Kimmo Lönnrot (Principal Investigator); Teppo Järvinen (Co-Principal Investigator); Simo Taimela (Co-Principal Investigator, Data Management) Pirjo Toivonen (Research Coordination).

*Central Adjudication Committee:* Kimmo Lönnrot, Ville Leinonen, Janek Frantzen.

*Data Safety Monitoring Board:* Kari Tikkinen (Chair), Christopf Schwartz, Timo Koivisto.

**FACADE Investigators**

The following persons participated in or have Ethics Board approval to participate in the FACADE trial at the time of submission of this manuscript: *Helsinki University Hospital* – Kimmo Lönnrot, Teppo Järvinen, Simo Taimela, Marja Silvasti-Lundell, Johannes Förster, Anniina Koski-Palken, Jarno Satopää, Behnam Rezai-Jahromi, Pasi Aronen, Pirjo Toivonen, Marketta Rautanen, Maarit Tuomisto.

**Funding**

This study was supported by the State funding for university-level health research (Helsinki University Hospitals). The funding source will have no role in the collection, analysis and

interpretation of data; in the writing of the report and in the decision to submit the article for publication

**Summary Competing Interests statement for all authors in the manuscript**

All authors have completed the ICMJE uniform disclosure form at www.icmje.org/coi_disclosure.pdf and declare: “No support from any commercial (industrial) organisation for the submitted work; no financial relationships with any organisations that might have an interest in the submitted work in the previous three years; no other relationships or activities that could appear to have influenced the submitted work.”

## REFERENCES


1. Radhakrishnan K, Litchy WJ, O'Fallon WM, Kurland LT. Epidemiology of cervical radiculopathy. Brain. 1994.
2. Cloward R. The anterior approach for removal of ruptured cervical disks. J Neurosurg. 1958;15:602–17.
3. Smith G, Robinson R. The treatment of certain cervical-spine disorders by anterior removal of the intervertebral disc and interbody fusion. J bone Jt Surg. 1958;40:607–24.
4. Albert TJ, Murrell SE. Surgical management of cervical radiculopathy. The Journal of the American Academy of Orthopaedic Surgeons. 1999.
5. Miller LE, Block JE. Safety and effectiveness of bone allografts in anterior cervical discectomy and fusion surgery. Spine (Phila Pa 1976). 2011;
6. Adamson T, Godil SS, Mehrlich M, Mendenhall S, Asher AL, McGirt MJ. Anterior cervical discectomy and fusion in the outpatient ambulatory surgery setting compared with the inpatient hospital setting: analysis of 1000 consecutive cases. J Neurosurg Spine. 2016;24:878–84.
7. Purger DA, Pendharkar A V., Ho AL, Sussman ES, Yang L, Desai M, et al. Outpatient vs inpatient anterior cervical discectomy and fusion: A population-level analysis of outcomes and cost. Clin Neurosurg. 2018;
8. McGirt MJ, Godil SS, Asher AL, Parker SL, Devin CJ. Quality analysis of anterior cervical discectomy and fusion in the outpatient versus inpatient setting: analysis of 7288 patients from the NSQIP database. Neurosurg Focus. 2015;
9. Mohandas A, Summa C, Worthington WB, Lerner J, Foley KT, Bohinski RJ, et al. Best Practices for Outpatient Anterior Cervical Surgery. Spine (Phila Pa 1976). 2017;
10. Piaggio G, Elbourne DR, Pocock SJ, Evans SJW, Altman DG. Reporting of noninferiority and equivalence randomized trials: extension of the CONSORT 2010 statement. JAMA. 2012 Dec;308(24):2594–604.
11. Vernon H, Mior S. The Neck Disability Index: a study of reliability and validity. J Manipulative Physiol Ther [Internet]. 1991 Sep [cited 2019 Apr 25];14(7):409–15. Available from: http://www.ncbi.nlm.nih.gov/pubmed/1834753
12. Westaway MD, Stratford PW, Binkley JM. The Patient-Specific Functional Scale: Validation of Its Use in Persons With Neck Dysfunction. J Orthop Sport Phys Ther [Internet]. 1998 May [cited 2019 Apr 25];27(5):331–8. Available from: http://www.ncbi.nlm.nih.gov/pubmed/9580892
13. Salo P, Ylinen J, Kautiainen H, Arkela-Kautiainen M, Häkkinen A. Reliability and validity of the finnish version of the neck disability index and the modified neck pain and disability scale. Spine (Phila Pa 1976) [Internet]. 2010 Mar 1 [cited 2018 Sep 16];35(5):552–6. Available from: https://insights.ovid.com/crossref?an=00007632-201003010-00014
14. Parker SL, Godil SS, Shau DN, Mendenhall SK, McGirt MJ. Assessment of the minimum clinically important difference in pain, disability, and quality of life after anterior cervical discectomy and fusion: clinical article. J Neurosurg Spine. 2013;
15. Froud R, Fawkes ; Carol, Foss J, Underwood M, Carnes D. Responsiveness, Reliability, and Minimally

Important and Minimal Detectable Changes of 3 Electronic Patient-Reported Outcome Measures for Low Back Pain: Validation Study. [cited 2019 Feb 8]; Available from: http://www.jmir.org/2018/10/e272/

16. Ahlstrom L, Grimby-Ekman A, Hagberg M, Dellve L. The work ability index and single-item question: Associations with sick leave, symptoms, and health - A prospective study of women on long-term sick leave. Scand J Work Environ Heal. 2010;

17. Stolk E, Ludwig K, Rand K, van Hout B, Ramos-Goñi JM. Overview, Update, and Lessons Learned From the International EQ-5D-5L Valuation Work: Version 2 of the EQ-5D-5L Valuation Protocol. Value Heal [Internet]. 2019 Jan 1 [cited 2019 Apr 25];22(1):23–30. Available from: https://www.sciencedirect.com/science/article/pii/S1098301518361643?via%3Dihub

18. Zoëga B, Kärrholm J, Lind B. Outcome scores in degenerative cervical disc surgery. Eur Spine J. 2000;

19. Odom GL, Finney W, Woodhall B. Cervical disk lesions. J Am Med Assoc [Internet]. 1958 Jan 4 [cited 2019 Mar 13];166(1):23–8. Available from: http://www.ncbi.nlm.nih.gov/pubmed/13491305

20. Rihn JA, Kane J, Albert TJ, Vaccaro AR, Hilibrand AS. What Is the Incidence and Severity of Dysphagia After Anterior Cervical Surgery? Clin Orthop Relat Res [Internet]. 2011 Mar 8 [cited 2019 Mar 17];469(3):658–65. Available from: http://www.ncbi.nlm.nih.gov/pubmed/21140251

21. Martins PC, Couto TE, Gama ACC. Auditory-perceptual evaluation of the degree of vocal deviation: correlation between the Visual Analogue Scale and Numerical Scale. CoDAS [Internet]. 2015 Jun [cited 2019 Mar 17];27(3):279–84. Available from: http://www.scielo.br/scielo.php?script=sci_arttext&pid=S2317-17822015000300279&lng=en&tlng=en

22. Audigier V, White IR, Jolani S, Debray TPA, Quartagno M, Carpenter J, et al. Multiple Imputation for Multilevel Data with Continuous and Binary Variables. Stat Sci [Internet]. 2018 [cited 2019 Mar 13];33(2):160–83. Available from: https://doi.org/10.1214/18-STS646

23. Sihvonen R, Paavola M, Malmivaara A, Itala A, Joukainen A, Nurmi H, et al. Arthroscopic partial meniscectomy versus sham surgery for a degenerative meniscal tear. N Engl J Med. 2013 Dec;369(26):2515–24.

24. Paavola M, Malmivaara A, Taimela S, Kanto K, Inkinen J, Kalske J, et al. Subacromial decompression versus diagnostic arthroscopy for shoulder impingement: randomised, placebo surgery controlled clinical trial. BMJ. 2018 Jul;362:k2860.

25. Jarvinen TLN, Sihvonen R, Bhandari M, Sprague S, Malmivaara A, Paavola M, et al. Blinded interpretation of study results can feasibly and effectively diminish interpretation bias. J Clin Epidemiol. 2014 Jul;67(7):769–72.

26. Richards T, Schroter S, Price A, Godlee F. Better together: patient partnership in medical journals. [cited 2019 Jan 11]; Available from: https://peerreviewcongress.org/prc17-0103

27. Piaggio G, Elbourne DR, Pocock SJ, Evans SJW, Altman DG, CONSORT Group for the. Reporting of Noninferiority and Equivalence Randomized Trials. JAMA [Internet]. 2012 Dec 26 [cited 2019 Feb 13];308(24):2594. Available from: http://jama.jamanetwork.com/article.aspx?doi=10.1001/jama.2012.87802

28. Etuknwa A, Daniels K, Eib · Constanze. Sustainable Return to Work: A Systematic Review Focusing on Personal and Social Factors. J Occup Rehabil [Internet]. [cited 2019 Sep 5];0. Available from: https://doi.org/10.1007/s10926-019-09832-7

# Statistical Analysis Plan and Blinded Data Interpretation Plan

Statistical analysis plan for Inpatient versus Outpatient Care after Anterior Cervical Decompression and Fusion: A prospective randomised non-inferiority trial

NCT03979443

August 16th 2022

We, the Blinded Data Interpretation Committee of the FACADE trial, have reached a consensus on how to carry out the blinded data interpretation (BDI). The document coined “Minutes for FACADE blinded data interpretation” (next page) outlines the execution of the blinded data interpretation for the FACADE trial.

Statistical analysis will be carried out by the trial statistician without any involvement from members of the Blinded Data Interpretation Committee or other FACADE investigators, as outlined below. The central study coordinator will code the trial data (two treatment arms) as ‘Group A’ and ‘Group B’ before handing the data over to the statistician. This will help ensure that the statistical analyses will be performed blind to the treatment allocation.

To reduce risk of interpretation bias, blinded results from the ITT analysis (Group A vs. Group B) will be presented to the Blinded Data Interpretation Committee. The Blinded Data Interpretation Committee will then contemplate on two alternative written interpretations, one where group A is the Inpatient care strategy and one where Group A is the Outpatient care strategy. Only after the Blinded Data Interpretation Committee has reached a consensus on the proper interpretation of the findings, the central study coordinator will unblind the treatment group allocation.

Also, as Drs. Lönnrot and Satopää were involved in the clinical care of the patients, they will recuse themselves from making any interpretations but are to take part in the blinded data interpretation meeting to answer potential questions regarding the execution of the trial.

Finally, the undersigned (members of the FACADE Blinded Data Interpretation Committee) agree that the minutes of the upcoming blinded data interpretation meeting will be emailed to an independent scientist for external review (comments/requests for clarification) before the final manuscript is submitted.

FACADE Blinded Data Interpretation Committee August 16, 2022

Teppo Järvinen, MD, PhD (Chair, co-PI of the FACADE)

Simo Taimela

Simo Taimela, MD, PhD (Co-Chair)

Tomasz Czuba, Trial statistician

Kimmo Lönnrot, MD, PhD (FACADE PI)

Minutes of the FACADE Blinded Data Interpretation meeting for Manuscript:

**Inpatient versus Outpatient Care after Anterior Cervical Decompression and Fusion: A prospective randomised non-inferiority trial**

FACADE Writing committee:

Kimmo Lönnrot[1,2], SimoTaimela[2], and Teppo Järvinen[2]

Independent statistician:

Tomasz Czuba[3]

FACADE investigators (complete list):

Kimmo Lönnrot, Simo Taimela, Jarno Satopää, Johannes Förster, Marja Silvast-Lundell, Ilkka Saarenpää, Mikko Kauppinen, Behnam Rezai-Jahromi, Janek Frantzen, Ville Leinonen, Anniina Koski-Palkén, Juri Kivelev, Jussi Antinheimo, Matti Seppälä, Miikka Korja, Leena Kivipelto, Mikko Pitkänen, Maarit Tuomisto, Pirjo Toivonen, Tomasz Czuba, Teppo Järvinen.

Author affiliations

1. Department of Neurosurgery, University of Helsinki and Helsinki University Hospital, Helsinki, Finland
2. Finnish Centre for Evidence-Based Orthopedics (FICEBO), Department of Orthopedics and Traumatology, University of Helsinki and Helsinki University Hospital, Helsinki, Finland
3. Department of Cardiology, Institute of Medicine, Gothenburg University and Sahlgrenska University Hospital, Gothenburg, Sweden

**Correspondence to:**

Dr. Kimmo Lönnrot, Department of Neurosurgery, Helsinki University Hospital, Töölö hospital, Topeliuksenkatu 5, Helsinki 00260, Finland, tel+358-50-4270270, kimmo.lonnrot@hus.fi

## Background and trial objectives

During the past decade, cervical spine procedures have increasingly been performed on an outpatient basis and retrospective database analyses have shown this to be feasible and safe. However, to our knowledge, no randomized controlled trial exists to compare the safety and efficacy/effectiveness of outpatient vs. inpatient care in patients undergoing anterior cervical decompression and fusion (ACDF) procedure.

We designed a randomized controlled study comparing outpatient and inpatient care in patients undergoing ACDF, with a primary objective to assess whether outpatient care is non-inferior to inpatient care with regards to the patients' perception of symptom relief (assessed by NDI, our primary efficacy outcome).

## Methods

### Trial design and oversight

In this three-center, stratified, block-randomized trial we randomized 104 patients after ACDF procedure to two treatment groups in a 1:1 ratio: A strategy of early (6-8 hours after procedure) discharge (Outpatient group) or a strategy of staying under hospital surveillance overnight (Inpatient group).

The full study protocol of the FACADE study has been published[1]. The participating centers and study group are listed in the Supplementary Appendix. The trial was designed and conducted by the FACADE investigators and the analyses were completed at the coordinating center. The trial protocol was approved by ethical review at the institutional review board of the Helsinki and Uusimaa Hospital District on June 6, 2019 (1540/2019) and duly registered at ClinicalTrials.gov (NCT03979443: https://clinicaltrials.gov/ct2/show/NCT03979443). All participants provided written informed consent. The trial was monitored by Clinical Research Unit of the Helsinki University Hospital (HYKSIN Institute), Helsinki, Finland. The writing committee of the FACADE trial vouch for the accuracy and completeness of the data, the fidelity of the trial to the protocol, and the complete reporting of adverse events. There was no industry involvement in the trial.

The FACADE trial was launched in the coordinating center (Helsinki) on June 12, 2019. The next trial center (Turku University Hospital) joined the trial on January 16, 2020 and the third (Oulu University Hospital) on January 23, 2020. Both Helsinki and Turku retained in the trial until patient recruitment was completed (February 12, 2021). However, due to Covid-19 situation, Oulu was able participate trial only until May 30, 2020.

### Participants

During the recruitment period of the trial, we screened all patients suffering from radiating arm pain referred to the study centers for trial eligibility (n=782). After being fully informed of the trial protocol, 104 eligible patients willing to participate (written informed consent) were randomized.

### Randomization and Blinding

After the surgery, all patients were taken to the recovery room for 2 to 3 hours for an immediate postoperative observation. When we had confirmed that the patients were fully conscious and co-operative, and immediate postoperative complications were

ruled out using a postoperative checklist, patients' final eligibility was confirmed. A member of the FACADE study group then randomized the patients either to the Outpatient group (discharge within the same day) or the Inpatient group (overnight surveillance at the hospital). The randomisation was a built-in property in the online electronic case report form (eCRF) system used in the trial (Granitics Ltd., Espoo, Finland). To minimise the risk of predicting the treatment assignment of the next eligible patient (to ensure concealment), we performed randomisation with variable block size (block size known only to the statistician with no involvement in the clinical care of the participants in the trial).

**Study Interventions**

*Outpatient group*
A ward nurse evaluated all patients allocated to the Outpatient group approximately 6-8 hours after surgery using a standardised FACADE discharge checklist. If the patient fulfilled all discharge criteria, he/she was instructed on how to deal with any concerns and was discharged. At discharge, we documented the time elapsed from operation and provided the patients with prescriptions to manage postoperative pain and an absence from work medical certificate for the first postoperative week.

*Inpatient group*
Patients allocated to inpatient care were kept in the hospital for surveillance overnight. A neurosurgeon on duty assessed whether patients were fit to be discharged on the 1$^{st}$ postoperative day. Identically to the Outpatient group, we documented the time elapsed from operation at discharge. We also provided the patients with prescriptions for postoperative pain management and an absence from work medical certificate for the first postoperative week.

**Outcome measures**

*Primary (efficacy) outcome measure*
Our primary outcome measure was the Neck Disability Index (NDI; scale 0 to 100, with higher scores indicating worse outcomes and more symptoms), a validated, neck-specific, patient reported measure of pain-related dysfunction[2,3]. We used a validated Finnish version of the NDI[4]. The primary assessment time point was six months. We also gathered the NDI at one and three months postoperatively, but this data was only intended to illustrate the trajectory of the treatment responses (Table 2). Our pre-defined threshold for minimal important difference (MID) of the primary outcome (NDI) was set at 17.3% (improvement from the baseline value), based on previous literature[5].

Details of all other outcome measures can be found in the protocol and the Supplementary Appendix.

## Sample size

Originally, the trial was powered to detect an MID in the NDI score between the two study groups. We set the MID for NDI (17.3%) as our margin of non-inferiority Δ based on the results by Parker et al.[5] Assuming no difference between treatment arms (ε = 0 in NDI score improvements), equal sample sizes (x=1, the SD 23%), a margin of non-inferiority Δ of 17.3%, one-sided 2.5% statistical significance criteria ($z_\alpha$ = 1.96) and 90% statistical power ($z_\beta$ =1,28), the required sample size was 44 patients per study

group. When also taking a dropout rate of 15% into account, the group size increased to 52 patients. Accordingly, we set the recruitment target at 104 patients.

However, while our statistician (TC) was preparing the plan for blinded data interpretation, he noticed that we had erroneously chosen an incorrect value for SD (in the study by Parker et al.[5], the mean improvement for NDI was 23.2 while the standard deviation (SD) was 19.7%). Keeping all other parameters constant, he recalculated the required sample size: The recruitment target turned out 29 subjects per group (without adjusting for dropouts) and 35 subjects per group (after adjusting for a 15% dropout rate), respectively.

## Statistical analysis

The trial is primarily designed to ascertain whether outpatient care is non-inferior to inpatient care, at 6 months after surgery, with NDI as the primary outcome. Only one primary analysis will be used to assess non-inferiority. At the 6-month time point, non-inferiority can be claimed if the lower limit of the CI (based on difference in means in the NDI) is greater than the MID in the primary comparison.

According to the CONSORT (Consolidated Standards of Reporting Trials) statement for non-inferiority and equivalence trials[6], secondary outcomes can be managed using either a superiority or equivalence framework. In our trial, all secondary outcomes will be assessed with an equivalence hypothesis, but since our trial is not necessarily powered for these comparisons, and to avoid issues with multiplicity, we consider them exploratory or hypothesis-generating.

We will follow primarily intention-to-treat (ITT) principle in all our analyses. In the ITT analyses, the participants are included as randomised. Per-protocol and on-treatment analyses will also be used to avoid falsely claiming non-inferiority. Summary statistics will be given as mean (with SD) for continuous variables and as frequencies (with %) for categorical variables. Repeated measures mixed model (RMMM) analysis will be used for all continuous variables (both primary and secondary outcomes) where regression coefficients are allowed to differ between study subjects. Statistical significance is set to two-sided 5% level. The RMMM analysis allows the use of all available observations in the data set, so the full data set (data set without multiple imputation) will be used in the analysis. Logistic regression will be used to assess categorical variables. STATA (Statistics/Data analysis, SE v15.1, StataCorp LLC, 4905 Lakeway Drive, College Station, Texas 77845 USA) will be used for analyses.

## Blinded data interpretation

The data will be interpreted according to a blinded data interpretation scheme we have published and described in detail previously.[5] In brief, FACADE statistician (TC) will carry out the statistical analyses, blinded to the group assignment, and presents the data as Group A and Group B. The FACADE Blinded Data Interpretation committee will then contemplate on the blinded results until a consensus on the interpretation is reached. Once the Blinded Data Interpretation committee reaches a consensus, the data will be unblinded and no changes are made to the interpretation of the results.

In keeping with the pre-defined interpretation plan for the FACADE trial, we will adhere to the following plan in presenting and interpreting the data (presented as Group A and Group B to preserve blinding) at the BDI meeting:

**1. Analysis on efficacy (primary, non-inferiority analysis): Is outpatient care non-inferior to inpatient care after anterior cervical decompression and fusion?**

- Table 1. Baseline characteristics.
- Table 2. Primary outcome (NDI) at the primary outcome assessment time point (6 mo).
- Figures 2 and 3: Two possible scenarios – Group A vs. Group B and Group B vs. Group A.

Based on this data, we will make an initial (blinded) interpretation on non-inferiority.

**2. Treatment-related adverse consequences of both treatment strategies (Safety concerns)**

Before finalising our interpretation on clinical relevance of our findings, we will assess the safety of the two treatment arms:

**Table 3.** Complications, adverse events, re-admissions to hospital

According to our own data on the safety of the ACDF procedure (overall complications rates < 10%), this analysis will likely not be powered to materially change our main conclusion, particularly with regards to the most feared complication (neck haematoma), which has a reported incidence of <1%.

Having said this, to be completely transparent and inclusive about the possible effect of adverse consequences on the clinical relevance (our interpretation) of the trial findings, we commit to assessing the overall rate (incidence) of safety concerns before final interpretation is made as follows:

If we detect > 10% difference in the overall incidence of serious adverse events between the two group (treatment strategies), we will add the following notion in our conclusion:

"*However, there was a noteworthy imbalance in the incidence of safety concerns in the two treatment strategies (higher/lower rate in Group A) and this should be considered when interpreting the trial findings.*"

As noted, the analysis on the downsides of the two treatments will not change our assessment **on efficacy**, rather modify our interpretation on the clinical relevance of the trial.

**The sequence of events to take place in the upcoming "blinded data interpretation meeting" is outlined in the flow chart below:**

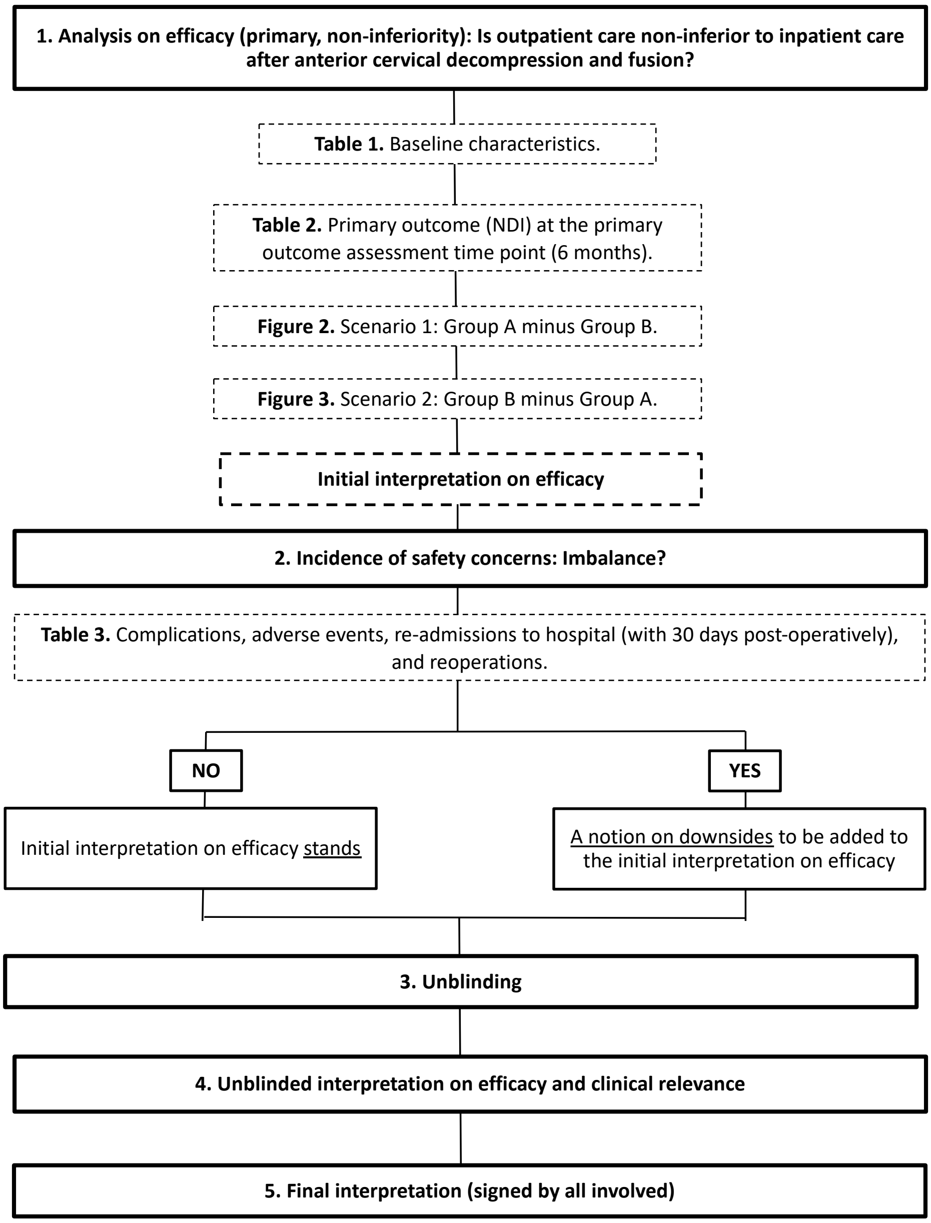
1. Analysis on efficacy (primary, non-inferiority): Is outpatient care non-inferior to inpatient care after anterior cervical decompression and fusion?
Table 1. Baseline characteristics.
Table 2. Primary outcome (NDI) at the primary outcome assessment time point (6 months).
Figure 2. Scenario 1: Group A minus Group B.
Figure 3. Scenario 2: Group B minus Group A.
Initial interpretation on efficacy
2. Incidence of safety concerns: Imbalance?
Table 3. Complications, adverse events, re-admissions to hospital (with 30 days post-operatively), and reoperations.
NO
YES
Initial interpretation on efficacy stands
A notion on downsides to be added to the initial interpretation on efficacy
3. Unblinding
4. Unblinded interpretation on efficacy and clinical relevance
5. Final interpretation (signed by all involved)

## 1. Analysis on efficacy (primary, non-inferiority): Is outpatient care non-inferior to inpatient care after anterior cervical decompression and fusion?

**Table 1.** Baseline characteristics.

| | Group A | | Group B | |
|---|---|---|---|---|
| | | Mean ± SD | | Mean ± SD |
| Age (years), mean (SD) | | | | |
| Gender (female), n | | | | |
| Dominant hand affected, n | | | | |
| Work Ability Score (WAS) | | | | |
| Physically demanding job: “heavy labor”, n | | | | |
| Patient’s own estimate of job demands: “heavy” n | | | | |
| Ability to work normally irrespective of the symptoms? n | | | | |
| Participation in leisure time activities irrespective of the symptoms? n | | | | |
| Duration of symptoms (days), mean (SD) | | | | |
| Preoperative sick leave (days) | | | | |
| Prior treatments (Physiotherapy) n | | | | |
| NSAID Pain medication, n | | | | |
| Opioid pain medication, n | | | | |
| Neuropathic pain medication, n | | | | |
| Neck Disability Index (NDI) (scale: 0 to 100), mean (SD) | | | | |
| Neck pain at rest (NRS scale: 0 to 10), mean (SD) | | | | |
| Arm pain at rest (NRS scale: 0 to 10), mean (SD) | | | | |
| EuroQol-5 dimensions-5 levels Time Trade-Off index score (0 to 1) | | | | |
| EuroQol-5 dimensions-5 levels Health Visual Analogue Scale (0 to 100) | | | | |

**Table 2.** Primary outcome at the primary outcome assessment time point (6 months).

| **Table 2. Primary outcome at six months** | | | | | |
|---|---|---|---|---|---|
| | **Group A** | | **Group B** | | **Difference (95% CI)** |
| | | Mean ± SE | | Mean ± SE | |
| **Primary efficacy outcome** | | | | | |
| Neck Disability Index (Scale: 0 to 100) | | ?? ± ?? | | ?? ± ?? | ?? ± ?? |
| ***Means and standard errors are derived from a general linear repeated measures model analysis.** **Abbreviations: CI: Confidence Interval; SE: Standard Error** | | | | | |

Our judgment on the efficacy (non-inferiority) will be based on the location of the whole CI in relation to Δ (non-inferiority margin), as outlined by Piaggio et al[6].

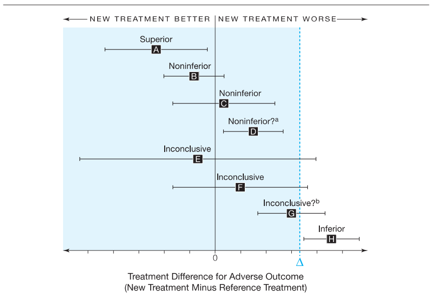


Error bars indicate 2-sided 95% CIs. The blue dashed line at x=Δ indicates the noninferiority margin; the blue tinted region to the left of x=Δ indicates the zone of inferiority. A, If the CI lies wholly to the left of zero, the new treatment is superior. B and C, If the CI lies to the left of Δ and includes zero, the new treatment is noninferior but not shown to be superior. D, If the CI lies wholly to the left of Δ and wholly to the right of zero, the new treatment is noninferior in the sense already defined but also inferior in the sense that a null treatment difference is excluded. This puzzling circumstance is rare, because it requires a very large sample size. It also can result from a noninferiority margin that is too wide. E and F, If the CI includes Δ and zero, the difference is nonsignificant but the result regarding noninferiority is inconclusive. G, If the CI includes Δ and is wholly to the right of zero, the difference is statistically significant but the result is inconclusive regarding possible inferiority of magnitude Δ or worse. H, If the CI is wholly above Δ, the new treatment is inferior.
[a] This CI indicates noninferiority in the sense that it does not include Δ, but the new treatment is significantly worse than the standard. Such a result is unlikely because it would require a very large sample size.
[b] This CI is inconclusive in that it is still plausible that the true treatment difference is less than Δ, but the new treatment is significantly worse than the standard. Adapted from Piaggio et al.[6]

As we will not have knowledge of treatment group assignment (whether Group A or Group B is our “new treatment”: here, Outpatient care), and to preserve our blinding, we have deemed it necessary to take both scenarios under consideration, as follows:

- We will calculate the treatment group difference assuming first that Group A is the “new treatment” and then that Group B is the “new treatment” (Scenario 1 and Scenario 2).
- We will plot the resulting point estimate with error bars (95% Cis) into two separate graphs.
- We will interpret both graphs (**Figures 2** and **3**).

**Figure 2.** Scenario 1: Group A minus Group B.

(EXAMPLE GRAPH BELOW, to be replaced by the actual graph of the FACADE trial data).

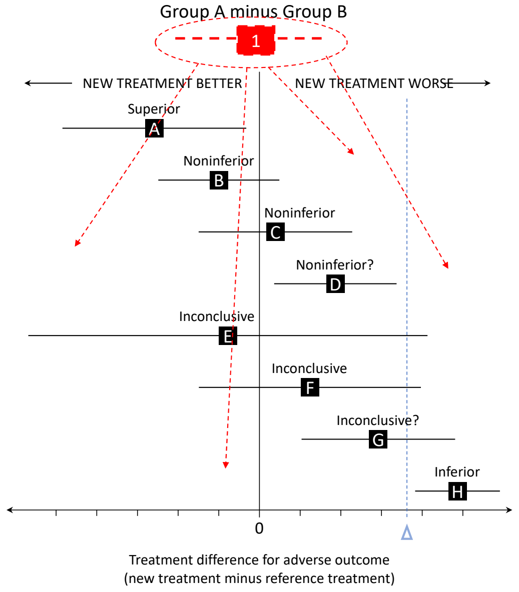


**Figure 3.** Scenario 2: Group B minus Group A.

(EXAMPLE GRAPH BELOW, to be replaced by the actual graph of the FACADE trial data).

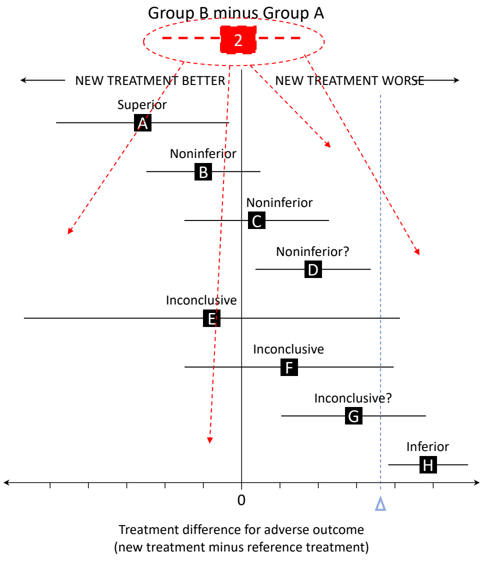

**Initial interpretation:**

Based on the location of the whole CI in relation to Δ (non-inferiority margin), our initial interpretation on the non-inferiority of Outpatient care (vs. Inpatient care), is as follows:

**Scenario 1 (Figure 2)** [incorrect options to be removed]

Group A is [ superior A / non-inferior B to D / inferior H ] to Group B after anterior cervical decompression and fusion.

OR

Our results are inconclusive [ E to G ] regarding the non-inferiority of the two groups after anterior cervical decompression and fusion.

**Scenario 2 (Figure 3)** [incorrect options to be removed]

Group B is [ superior A / non-inferior B to D / inferior H ] to Group A after anterior cervical decompression and fusion.

OR

Our results are inconclusive [ E to G ] regarding the non-inferiority of the two groups after anterior cervical decompression and fusion.

## 2. Safety concerns

**Table 3.** Complications, adverse events, re-admissions to hospital (with 30 days post-operatively), and reoperations.

| | Group A | Group B |
|---|---|---|
| | | |
| **Acute perioperative complications** | n | n |
| | | |
| **Serious Adverse Events (SAE)** | Below % will represent % of SAE/MAE/Cause of reop in group A not % of total N | Below % will represent % of SAE in group B not % of total N |
| Cardiovascular event | n (%) | n (%) |
| Pulmonary embolus | n (%) | n (%) |
| Deep venous thrombosis in leg | n (%) | n (%) |
| Subcutaneus neck haematoma | n (%) | n (%) |
| Systemic infection | n (%) | n (%) |
| Postoperative hemi- or tetraplegia | n (%) | n (%) |
| Persistent dysphonia at 6 months | n (%) | n (%) |
| Persistent dysphagia at 6 months | n (%) | n (%) |
| Death | n (%) | n (%) |
| Other | n (%) | n (%) |
| | | |
| **Minor Adverse Events (MAE)** | | |
| Wound infection | n (%) | n (%) |
| Motor deficit (new) | n (%) | n (%) |
| Persistent symptoms | n (%) | n (%) |
| Dyspnea (return to hospital) | n (%) | n (%) |
| | | |
| **Re-admissions to hospital (< 30 days)** | n (%) | n (%) |
| | | |
| **Cause of reoperation** | | |
| Impaction of implant | n (%) | n (%) |
| Dislocation of implant | n (%) | n (%) |
| Foraminal re-stenosis | n (%) | n (%) |
| Wound infection | n (%) | n (%) |
| Wound opening | n (%) | n (%) |
| Other cause | n (%) | n (%) |

**Imbalance between the two groups in the crude incidence of safety concerns?**

YES / NO

- If NO, our initial interpretation on efficacy (previous page) stands as is.
- If YES => We will add the following notion about the imbalance (excess in harms) to our interpretation:

"*However, there was a noteworthy imbalance in the incidence of safety concerns in the two treatment strategies (higher/lower rate in Group A) and this should be considered when interpreting the trial findings.*"

**3. Unblinding**
After consideration of the major downsides of the two treatment groups, we have now reached a consensus on our blinded assessment on efficacy.

Our statistician will now unblind the treatment group assignment (break the randomization code):

Group A = Inpatient care / Outpatient care [incorrect option to be removed]
Group B = Inpatient care / Outpatient care [incorrect option to be removed]

Given the above noted, the FACADE data is shown in **Table 1** (with n-values for Groups to be added) and in Scenario 1 (**Figure 2**) or Scenario 2 (**Figure 3**). [incorrect option to be removed]

**Table 2.** Primary outcome at the primary outcome assessment time point (6 months).

| Table 2. Primary outcome at six months | | | | | |
|---|---|---|---|---|---|
| | **Group A** | | **Group B** | | **Difference (95% CI)** |
| | | Mean ± SE | | Mean ± SE | |
| **Primary efficacy outcome** | | | | | |
| Neck Disability Index (Scale: 0 to 100) | | ?? ± ?? | | ?? ± ?? | ?? ± ?? |
| ***Means and standard errors are derived from a general linear repeated measures model analysis.** **Abbreviations: CI: Confidence Interval; SE: Standard Error** | | | | | |

**Figure 2** or **Figure 3**

**4. Unblinded interpretation on efficacy and clinical relevance**

Accordingly, our interpretation of the FACADE trial is as follows:

[incorrect options to be removed]

(1) Inpatient care is superior to outpatient care after anterior cervical decompression and fusion. However, be it noted that we did not set a superiority hypothesis in the study protocol.

OR

(2) Inpatient care is non-inferior to outpatient care after anterior cervical decompression and fusion.

OR

(3) Inpatient care is inferior to outpatient care after anterior cervical decompression and fusion.

OR

(4) Our results are inconclusive regarding the non-inferiority of the two groups after anterior cervical decompression and fusion.

In addition to the primary conclusion above, the following notion regarding downsides of the two treatments will / will not be added [incorrect option to be removed] based on our assessment of the need for safety concerns (Section 2):

"However, there was a noteworthy imbalance in the safety of the two treatment strategies (higher/lower [incorrect option to be removed] rate in Outpatient care) and this should be considered when interpreting the trial findings."

**5. Final interpretation**
Our final interpretation of the FACADE trial stands as follows:

[Copy & paste the correct interpretation]

Place: ZOOM-/Teams-meeting
Time: [Insert date here]

______________________________
Teppo Järvinen

______________________________
Simo Taimela

______________________________
Tomasz Czuba, trial statistician

**Also present at the meeting (as external observers):**

______________________________
Kimmo Lönnrot, MD, PhD

______________________________
Rahul Raj, MD, PhD

______________________________
Jarno Satopää, MD, PhD

# Minutes of the Statistical Analysis Plan and Blinded Data Interpretation

Minutes of the FACADE Blinded Data Interpretation meeting for Manuscript:

**Inpatient versus Outpatient Care after Anterior Cervical Decompression and Fusion: A prospective randomised non-inferiority trial**


FACADE Writing committee:

Kimmo Lönnrot[1,2], SimoTaimela[2], and Teppo Järvinen[2]

Independent statistician:

Tomasz Czuba[3]

FACADE investigators (complete list):

Kimmo Lönnrot, Simo Taimela, Jarno Satopää, Johannes Förster, Marja Silvast-Lundell, Ilkka Saarenpää, Mikko Kauppinen, Behnam Rezai-Jahromi, Janek Frantzen, Ville Leinonen, Anniina Koski-Palkén, Juri Kivelev, Jussi Antinheimo, Matti Seppälä, Miikka Korja, Leena Kivipelto, Mikko Pitkänen, Maarit Tuomisto, Pirjo Toivonen, Tomasz Czuba, Teppo Järvinen.

Author affiliations

1. Department of Neurosurgery, University of Helsinki and Helsinki University Hospital, Helsinki, Finland
2. Finnish Centre for Evidence-Based Orthopedics (FICEBO), Department of Orthopedics and Traumatology, University of Helsinki and Helsinki University Hospital, Helsinki, Finland
3. Department of Cardiology, Institute of Medicine, Gothenburg University and Sahlgrenska University Hospital, Gothenburg, Sweden

**Correspondence to:**

Dr. Kimmo Lönnrot, Department of Neurosurgery, Helsinki University Hospital, Töölö hospital, Topeliuksenkatu 5, Helsinki 00260, Finland, tel+358-50-4270270, kimmo.lonnrot@hus.fi

## Background and trial objectives

During the past decade, cervical spine procedures have increasingly been performed on an outpatient basis and retrospective database analyses have shown this to be feasible and safe. However, to our knowledge, no randomized controlled trial exists to compare the safety and efficacy/effectiveness of outpatient vs. inpatient care in patients undergoing anterior cervical decompression and fusion (ACDF) procedure.

We designed a randomized controlled study comparing outpatient and inpatient care in patients undergoing ACDF, with a primary objective to assess whether outpatient care is non-inferior to inpatient care with regards to the patients' perception of symptom relief (assessed by NDI, our primary efficacy outcome).

## Methods

### Trial design and oversight

In this three-center, stratified, block-randomized trial we randomized 104 patients after ACDF procedure to two treatment groups in a 1:1 ratio: A strategy of early (6-8 hours after procedure) discharge (Outpatient group) or a strategy of staying under hospital surveillance overnight (Inpatient group).

The full study protocol of the FACADE study has been published[1]. The participating centers and study group are listed in the Supplementary Appendix. The trial was designed and conducted by the FACADE investigators and the analyses were completed at the coordinating center. The trial protocol was approved by ethical review at the institutional review board of the Helsinki and Uusimaa Hospital District on June 6, 2019 (1540/2019) and duly registered at ClinicalTrials.gov (NCT03979443: https://clinicaltrials.gov/ct2/show/NCT03979443). All participants provided written informed consent. The trial was monitored by Clinical Research Unit of the Helsinki University Hospital (HYKSIN Institute), Helsinki, Finland. The writing committee of the FACADE trial vouch for the accuracy and completeness of the data, the fidelity of the trial to the protocol, and the complete reporting of adverse events. There was no industry involvement in the trial.

The FACADE trial was launched in the coordinating center (Helsinki) on June 12, 2019. The next trial center (Turku University Hospital) joined the trial on January 16, 2020 and the third (Oulu University Hospital) on January 23, 2020. Both Helsinki and Turku retained in the trial until patient recruitment was completed (February 12, 2021). However, due to Covid-19 situation, Oulu was able participate trial only until May 30, 2020.

### Participants

During the recruitment period of the trial, we screened all patients suffering from radiating arm pain referred to the study centers for trial eligibility (n=782). After being fully informed of the trial protocol, 104 eligible patients willing to participate (written informed consent) were randomized.

### Randomization and Blinding

After the surgery, all patients were taken to the recovery room for 2 to 3 hours for an immediate postoperative observation. When we had confirmed that the patients were fully conscious and co-operative, and immediate postoperative complications were

ruled out using a postoperative checklist, patients' final eligibility was confirmed. A member of the FACADE study group then randomized the patients either to the Outpatient group (discharge within the same day) or the Inpatient group (overnight surveillance at the hospital). The randomisation was a built-in property in the online electronic case report form (eCRF) system used in the trial (Granitics Ltd., Espoo, Finland). To minimise the risk of predicting the treatment assignment of the next eligible patient (to ensure concealment), we performed randomisation with variable block size (block size known only to the statistician with no involvement in the clinical care of the participants in the trial).

**Study Interventions**

*Outpatient group*
A ward nurse evaluated all patients allocated to the Outpatient group approximately 6-8 hours after surgery using a standardised FACADE discharge checklist. If the patient fulfilled all discharge criteria, he/she was instructed on how to deal with any concerns and was discharged. At discharge, we documented the time elapsed from operation and provided the patients with prescriptions to manage postoperative pain and an absence from work medical certificate for the first postoperative week.

*Inpatient group*
Patients allocated to inpatient care were kept in the hospital for surveillance overnight. A neurosurgeon on duty assessed whether patients were fit to be discharged on the 1st postoperative day. Identically to the Outpatient group, we documented the time elapsed from operation at discharge. We also provided the patients with prescriptions for postoperative pain management and an absence from work medical certificate for the first postoperative week.

**Outcome measures**

*Primary (efficacy) outcome measure*
Our primary outcome measure was the Neck Disability Index (NDI; scale 0 to 100, with higher scores indicating worse outcomes and more symptoms), a validated, neck-specific, patient reported measure of pain-related dysfunction[2,3]. We used a validated Finnish version of the NDI[4]. The primary assessment time point was six months. We also gathered the NDI at one and three months postoperatively, but this data was only intended to illustrate the trajectory of the treatment responses (Table 2). Our pre-defined threshold for minimal important difference (MID) of the primary outcome (NDI) was set at 17.3% (improvement from the baseline value), based on previous literature[5].

Details of all other outcome measures can be found in the protocol and the Supplementary Appendix.

## Sample size

Originally, the trial was powered to detect an MID in the NDI score between the two study groups. We set the MID for NDI (17.3%) as our margin of non-inferiority Δ based on the results by Parker et al.[5] Assuming no difference between treatment arms (ε = 0 in NDI score improvements), equal sample sizes (x=1, the SD 23%), a margin of non-inferiority Δ of 17.3%, one-sided 2.5% statistical significance criteria ($z_\alpha$ = 1.96) and 90% statistical power ($z_\beta$ =1,28), the required sample size was 44 patients per study

group. When also taking a dropout rate of 15% into account, the group size increased to 52 patients. Accordingly, we set the recruitment target at 104 patients.

However, while our statistician (TC) was preparing the plan for blinded data interpretation, he noticed that we had erroneously chosen an incorrect value for SD (in the study by Parker et al.[5], the mean improvement for NDI was 23.2 while the standard deviation (SD) was 19.7%). Keeping all other parameters constant, he recalculated the required sample size: The recruitment target turned out 29 subjects per group (without adjusting for dropouts) and 35 subjects per group (after adjusting for a 15% dropout rate), respectively.

## Statistical analysis

The trial is primarily designed to ascertain whether outpatient care is non-inferior to inpatient care, at 6 months after surgery, with NDI as the primary outcome. Only one primary analysis will be used to assess non-inferiority. At the 6-month time point, non-inferiority can be claimed if the lower limit of the CI (based on difference in means in the NDI) is greater than the MID in the primary comparison.

According to the CONSORT (Consolidated Standards of Reporting Trials) statement for non-inferiority and equivalence trials[6], secondary outcomes can be managed using either a superiority or equivalence framework. In our trial, all secondary outcomes will be assessed with an equivalence hypothesis, but since our trial is not necessarily powered for these comparisons, and to avoid issues with multiplicity, we consider them exploratory or hypothesis-generating.

We will follow primarily intention-to-treat (ITT) principle in all our analyses. In the ITT analyses, the participants are included as randomised. Per-protocol and on-treatment analyses will also be used to avoid falsely claiming non-inferiority. Summary statistics will be given as mean (with SD) for continuous variables and as frequencies (with %) for categorical variables. Repeated measures mixed model (RMMM) analysis will be used for all continuous variables (both primary and secondary outcomes) where regression coefficients are allowed to differ between study subjects. Statistical significance is set to two-sided 5% level. The RMMM analysis allows the use of all available observations in the data set, so the full data set (data set without multiple imputation) will be used in the analysis. Logistic regression will be used to assess categorical variables. STATA (Statistics/Data analysis, SE v15.1, StataCorp LLC, 4905 Lakeway Drive, College Station, Texas 77845 USA) will be used for analyses.

## Blinded data interpretation

The data will be interpreted according to a blinded data interpretation scheme we have published and described in detail previously.[5] In brief, FACADE statistician (TC) will carry out the statistical analyses, blinded to the group assignment, and presents the data as Group A and Group B. The FACADE Blinded Data Interpretation committee will then contemplate on the blinded results until a consensus on the interpretation is reached. Once the Blinded Data Interpretation committee reaches a consensus, the data will be unblinded and no changes are made to the interpretation of the results.

In keeping with the pre-defined interpretation plan for the FACADE trial, we will adhere to the following plan in presenting and interpreting the data (presented as Group A and Group B to preserve blinding) at the BDI meeting:

**1. Analysis on efficacy (primary, non-inferiority analysis): Is outpatient care non-inferior to inpatient care after anterior cervical decompression and fusion?**

- Table 1. Baseline characteristics.
- Table 2. Primary outcome (NDI) at the primary outcome assessment time point (6 mo).
- Figures 2 and 3: Two possible scenarios – Group A vs. Group B and Group B vs. Group A.

Based on this data, we will make an initial (blinded) interpretation on non-inferiority.

**2. Treatment-related adverse consequences of both treatment strategies (Safety concerns)**

Before finalising our interpretation on clinical relevance of our findings, we will assess the safety of the two treatment arms:

**Table 3.** Complications, adverse events, re-admissions to hospital

According to our own data on the safety of the ACDF procedure (overall complications rates < 10%), this analysis will likely not be powered to materially change our main conclusion, particularly with regards to the most feared complication (neck haematoma), which has a reported incidence of <1%.

Having said this, to be completely transparent and inclusive about the possible effect of adverse consequences on the clinical relevance (our interpretation) of the trial findings, we commit to assessing the overall rate (incidence) of safety concerns before final interpretation is made as follows:

If we detect > 10% difference in the overall incidence of serious adverse events between the two group (treatment strategies), we will add the following notion in our conclusion:

"*However, there was a noteworthy imbalance in the incidence of safety concerns in the two treatment strategies (higher/lower rate in Group A) and this should be considered when interpreting the trial findings.*"

As noted, the analysis on the downsides of the two treatments will not change our assessment **on efficacy**, rather modify our interpretation on the clinical relevance of the trial.

**The sequence of events to take place in the upcoming "blinded data interpretation meeting" is outlined in the flow chart below:**

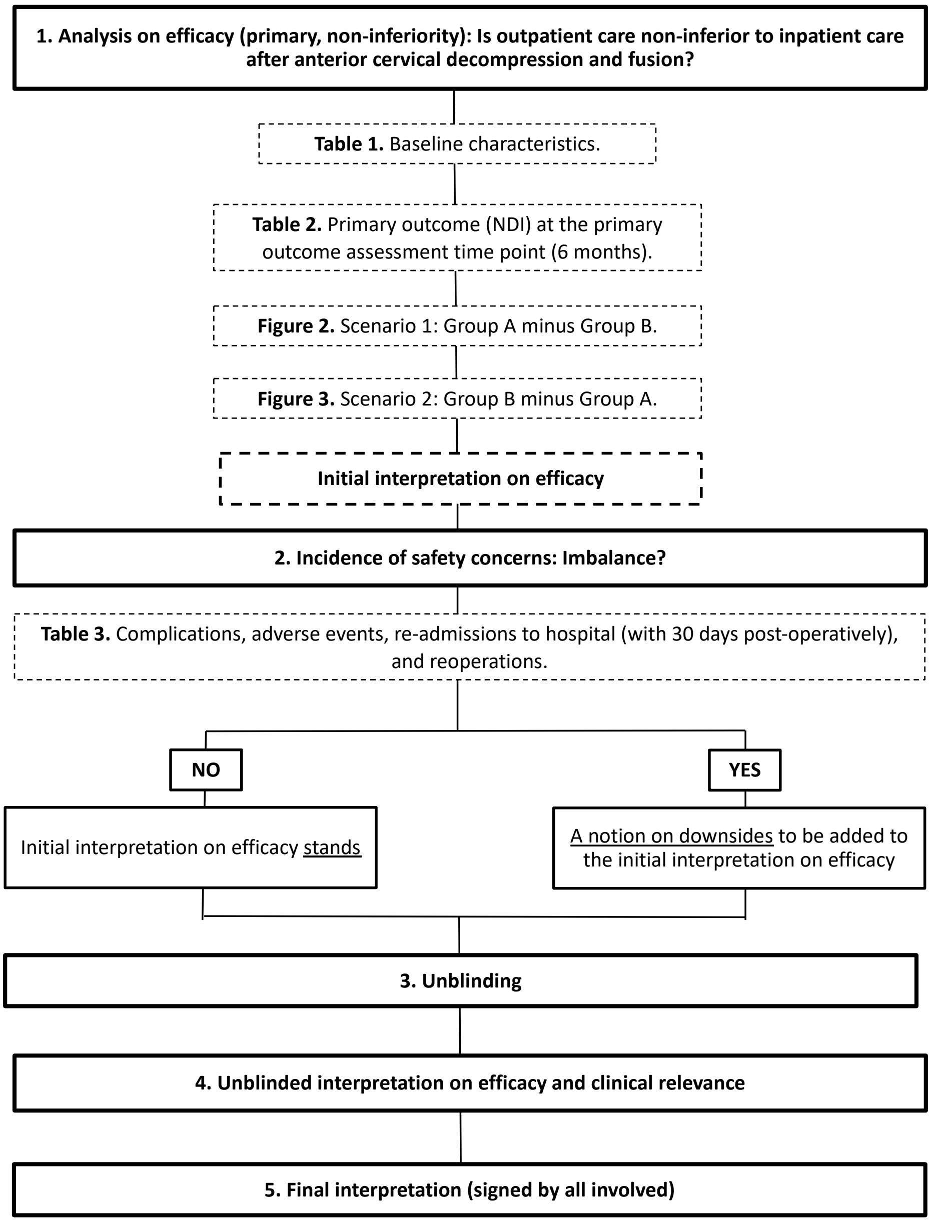

1. Analysis on efficacy (primary, non-inferiority): Is outpatient care non-inferior to inpatient care after anterior cervical decompression and fusion?
Table 1. Baseline characteristics.
Table 2. Primary outcome (NDI) at the primary outcome assessment time point (6 months).
Figure 2. Scenario 1: Group A minus Group B.
Figure 3. Scenario 2: Group B minus Group A.
Initial interpretation on efficacy
2. Incidence of safety concerns: Imbalance?
Table 3. Complications, adverse events, re-admissions to hospital (with 30 days post-operatively), and reoperations.
NO
YES
Initial interpretation on efficacy stands
A notion on downsides to be added to the initial interpretation on efficacy
3. Unblinding
4. Unblinded interpretation on efficacy and clinical relevance
5. Final interpretation (signed by all involved)

**1. Analysis on efficacy (primary, non-inferiority): Is outpatient care non-inferior to inpatient care after anterior cervical decompression and fusion?**

**Table 1.** Baseline characteristics.

| | Group A | | Group B | |
|---|---|---|---|---|
| | | Mean ± SD | | Mean ± SD |
| Age (years), mean (SD) | | 46.74 (7.34) | | 47.25 (8.39) |
| Gender (female), n | | 25 | | 28 |
| Dominant hand affected, n | | 21 | | 19 |
| Work Ability Score (WAS) | | 4.38 (2.20) | | 3.85 (2.42) |
| Physically demanding job: "heavy labor", n | | 31 | | 30 |
| Patient's own estimate of job demands: "heavy" n | | 31 | | 30 |
| Ability to work normally irrespective of the symptoms? n | | 17 | | 29 |
| Duration of symptoms (days), mean (SD) | | 261.18 (297.76) | | 370.86 (700.61) |
| Preoperative sick leave (days) | | 34.14 (30.40) | | 24.79 (30.81) |
| Prior treatments (Physiotherapy) n | | 31 | | 36 |
| NSAID Pain medication, n | | 39 | | 37 |
| Weak opioid pain medication, n | | 23 | | 17 |
| Strong opioid pain medication, n | | 0 | | 1 |
| Neuropathic pain medication, n | | 24 | | 24 |
| Neck Disability Index (NDI) (scale: 0 to 100), mean (SD) | | 48.15 (11.90) | | 45.08 (9.61) |
| Neck pain at rest (NRS scale: 0 to 10), mean (SD) | | 5.78 (1.97) | | 5.23 (2.09) |
| Arm pain at rest (NRS scale: 0 to 10), mean (SD) | | 6.35 (1.88) | | 6.67 (1.69) |
| EuroQol-5 dimensions-5 levels Time Trade-Off index score (0 to 1) | | 0.76 (0.10) | | 0.75 (0.08) |
| EuroQol-5 dimensions-5 levels Health Visual Analogue Scale (0 to 100) | | 54.46 (15.85) | | 53.44 (15.72) |

**Table 2.** Primary outcome at the primary outcome assessment time point (6 months).

| **Table 2. Primary outcome at six months** | | | | | |
|---|---|---|---|---|---|
| | **Group A** | | **Group B** | | **Difference (A – B) (95% CI)** |
| | | Mean ± SE | | Mean ± SE | |
| **Primary efficacy outcome** | | | | | |
| Neck Disability Index (Scale: 0 to 100) | | 13.33 ± 2.04 | | 12.23 ± 2.04 | 1.10 (-4.62 to 6.82) |
| ***Means and standard errors are derived from a general linear repeated measures model analysis.<br>Abbreviations: CI: Confidence Interval; SE: Standard Error** | | | | | |

Our judgment on the efficacy (non-inferiority) will be based on the location of the whole CI in relation to Δ (non-inferiority margin), as outlined by Piaggio et al[6].

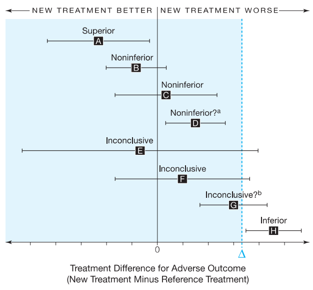


Error bars indicate 2-sided 95% CIs. The blue dashed line at x=Δ indicates the noninferiority margin; the blue tinted region to the left of x=Δ indicates the zone of inferiority. A, If the CI lies wholly to the left of zero, the new treatment is superior. B and C, If the CI lies to the left of Δ and includes zero, the new treatment is noninferior but not shown to be superior. D, If the CI lies wholly to the left of Δ and wholly to the right of zero, the new treatment is noninferior in the sense already defined but also inferior in the sense that a null treatment difference is excluded. This puzzling circumstance is rare, because it requires a very large sample size. It also can result from a noninferiority margin that is too wide. E and F, If the CI includes Δ and zero, the difference is nonsignificant but the result regarding noninferiority is inconclusive. G, If the CI includes Δ and is wholly to the right of zero, the difference is statistically significant but the result is inconclusive regarding possible inferiority of magnitude Δ or worse. H, If the CI is wholly above Δ, the new treatment is inferior.
[a] This CI indicates noninferiority in the sense that it does not include Δ, but the new treatment is significantly worse than the standard. Such a result is unlikely because it would require a very large sample size.
[b] This CI is inconclusive in that it is still plausible that the true treatment difference is less than Δ, but the new treatment is significantly worse than the standard. Adapted from Piaggio et al.[6]

As we will not have knowledge of treatment group assignment (whether Group A or Group B is our "new treatment": here, Outpatient care), and to preserve our blinding, we have deemed it necessary to take both scenarios under consideration, as follows:

- We will calculate the treatment group difference assuming first that Group A is the "new treatment" and then that Group B is the "new treatment" (Scenario 1 and Scenario 2).
- We will plot the resulting point estimate with error bars (95% Cis) into two separate graphs.
- We will interpret both graphs (**Figures 2** and **3**).

**Figure 2.** Scenario 1: Group A minus Group B.

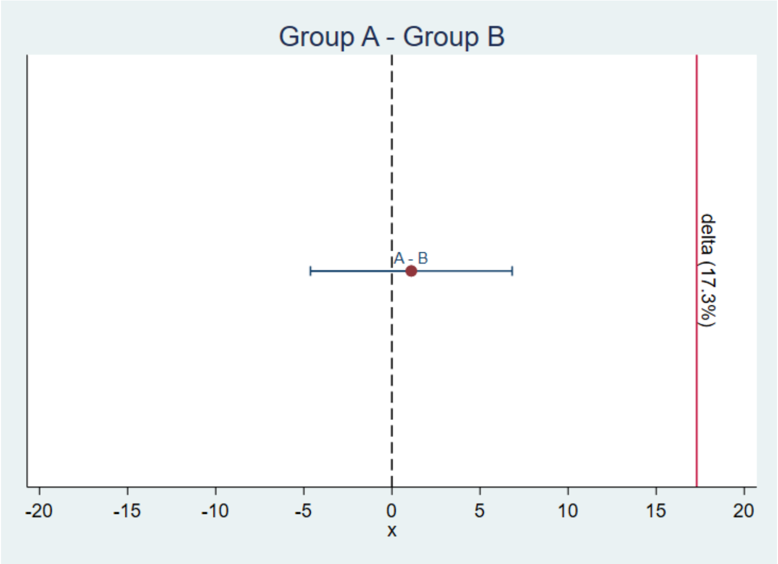


**Figure 3.** Scenario 2: Group B minus Group A.

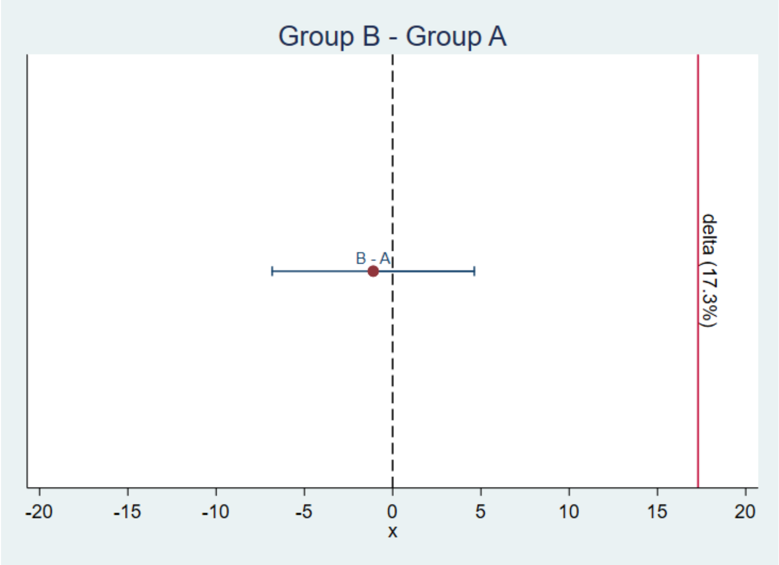

**Initial interpretation:**

Based on the location of the whole CI in relation to $\Delta$ (non-inferiority margin), our initial interpretation on the non-inferiority of Outpatient care (vs. Inpatient care), is as follows:

**Scenario 1 (Figure 2)**

Group A is non-inferior to Group B after anterior cervical decompression and fusion.

**Scenario 2 (Figure 3)**

Group B is non-inferior to Group A after anterior cervical decompression and fusion.

## 2. Safety concerns

**Table 3.** Complications, adverse events, re-admissions to hospital (with 30 days post-operatively), and reoperations.

| | Group A | Group B |
|---|---|---|
| | | |
| **Acute perioperative complications** | 1 | 1 |
| | | |
| **Serious Adverse Events (SAE)** | Below % will represent % of SAE/MAE/Cause of reop in group A not % of total N | Below % will represent % of SAE in group B not % of total N |
| Cardiovascular event | | |
| Pulmonary embolus | | |
| Deep venous thrombosis in leg | 0 | 1 (100.0) |
| Subcutaneus neck haematoma | | |
| Systemic infection | | |
| Postoperative hemi- or tetraplegia | | |
| Persistent dysphonia at 6 months | | |
| Persistent dysphagia at 6 months | | |
| Death | | |
| Other | | |
| | | |
| **Minor Adverse Events (MAE)** | | |
| Local infection | 3 (42.9) | 0 |
| Motor deficit (new) | | |
| Persistent symptoms | | |
| Dyspnea (return to hospital) | | |
| **transient dysphagia** | 2 (28.6) | 0 |
| **other infection** | 2 (28.6) | 0 |
| | | |
| **Re-admissions to hospital (< 30 days) due to adverse events** | n (%) | n (%) |
| **No** | 2 (28.6) | 1 (100.0) |
| **Yes** | 5 (71.4) | 0 |
| | | |
| **Cause of reoperation** | | |
| Impaction of implant | | |
| Dislocation of implant | | |
| Foraminal re-stenosis | 1 (100.0) | 0 |
| Wound infection | | |
| Wound opening | | |
| Other cause | | |

**Imbalance (>10% difference in SAE) between the two groups in the crude incidence of safety concerns?**

**NO**

- **If NO, our initial interpretation on efficacy (previous page) stands as is.**

- If YES => We will add the following notion about the imbalance (excess in harms) to our interpretation:

"*However, there was a noteworthy imbalance in the incidence of safety concerns in the two treatment strategies (higher/lower rate in Group A) and this should be considered when interpreting the trial findings.*"

**Notion by the blinded data interpreters:**

We note that the incidence of minor infections appears higher in group A. There are two alternative interpretations for this finding: This can be attributed to either early discharge (patients inability to maintain proper hygiene out of hospital) or hospital care increasing the risk of infections. Having said that, there was no discernible difference in the incidence of SAE and the overall incidence of safety concerns was in line with previously reported rates of adverse events after ACDF (e.g Kamalapathy PN, Puvanesarajah V, Sequeria S, Bell J, Hassanzadeh H. Safety profile of outpatient vs inpatient ACDF: An analysis of 33,807 outpatient ACDFs. Clin Neurol Neurosurg. 2021 Aug;207:106743. doi: 10.1016/j.clineuro.2021.106743. Epub 2021 Jun 8. PMID: 34153778.)

**3. Unblinding**
After consideration of the major downsides of the two treatment groups, we have now reached a consensus on our blinded assessment on efficacy.

Our statistician will now unblind the treatment group assignment (break the randomization code):

Group A = Outpatient care
Group B = Inpatient care

Given the above noted, the FACADE data is shown in **Table 1** (with n-values for Groups added) and in Scenario 1 (**Figure 2**).

**Table 2.** Primary outcome at the primary outcome assessment time point (6 months).

| Table 2. Primary outcome at six months | | | | | |
|---|---|---|---|---|---|
| | **Outpatient (n=52)** | | **Inpatient (n=52)** | | **Difference (95% CI)** |
| | | Mean ± SE | | Mean ± SE | |
| **Primary efficacy outcome** | | | | | |
| Neck Disability Index (Scale: 0 to 100) | | 13.33 ± 2.04 | | 12.23 ± 2.04 | 1.10 (-4.62 to 6.82) |
| ***Means and standard errors are derived from a general linear repeated measures model analysis.** | | | | | |
| **Abbreviations: CI: Confidence Interval; SE: Standard Error** | | | | | |

**Figure 2**

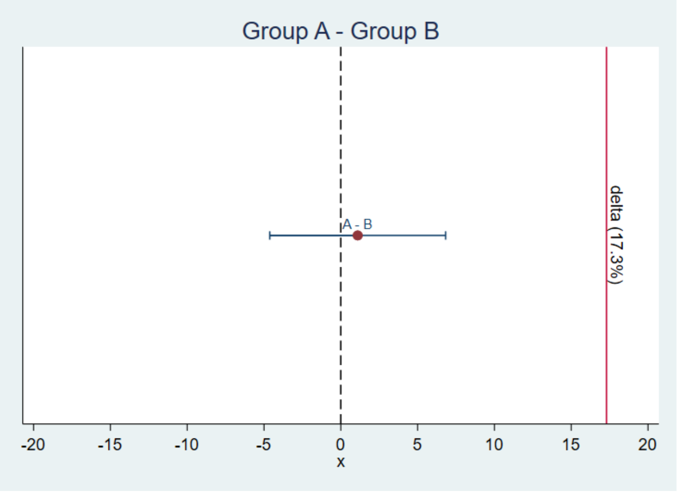

**4. Unblinded interpretation on efficacy and clinical relevance**

Accordingly, our interpretation of the FACADE trial is as follows:

Outpatient care is non-inferior to inpatient care after anterior cervical decompression and fusion.

**5. Final interpretation**
Our final interpretation of the FACADE trial stands as follows:

Outpatient care is non-inferior to inpatient care after anterior cervical decompression and fusion.

Place: ZOOM-meeting
Time: 22-08-2022

____________________________
Teppo Järvinen

Simo Taimela
____________________________
Simo Taimela

Tomasz Czuba
____________________________
Tomasz Czuba, trial statistician

**Also present at the meeting (as external observers):**

____________________________
Kimmo Lönnrot, MD, PhD

____________________________
Rahul Raj, MD, PhD

____________________________

**Signature:** Tomasz Czuba
Tomasz Czuba (Aug 22, 2022 18:05 GMT+2)

**Email:** toczuba@wp.pl

# minutes FACADE BDI

Final Audit Report 2022-08-22

| | |
|---|---|
| Created: | 2022-08-22 |
| By: | Simo Taimela (simo.taimela@evalua.fi) |
| Status: | Signed |
| Transaction ID: | CBJCHBCAABAAqYJ9SsprpkjLqJI9kO66AK-RhTxB4HeC |

## "minutes FACADE BDI" History

Document created by Simo Taimela (simo.taimela@evalua.fi)

2022-08-22 - 4:04:00 PM GMT- IP address: 85.156.237.7

Document emailed to toczuba@wp.pl for signature

2022-08-22 - 4:04:25 PM GMT

Email viewed by toczuba@wp.pl

2022-08-22 - 4:04:36 PM GMT- IP address: 193.222.135.162

Signer toczuba@wp.pl entered name at signing as Tomasz Czuba

2022-08-22 - 4:05:50 PM GMT- IP address: 83.254.141.159

Document e-signed by Tomasz Czuba (toczuba@wp.pl)

Signature Date: 2022-08-22 - 4:05:51 PM GMT - Time Source: server- IP address: 83.254.141.159

Agreement completed.

2022-08-22 - 4:05:51 PM GMT

Adobe Acrobat Sign
