# Supplementary material for: Ambulatory Care vs Overnight Hospitalization After Anterior Surgery for Cervical Radiculopathy: The FACADE Randomized Clinical Trial
Source: JAMA Netw Open. 2024 Nov 27;7(11):e2447459. doi: 10.1001/jamanetworkopen.2024.47459 (PMC12124692; doi:10.1001/jamanetworkopen.2024.47459)
Supplement: Supplement 2. — eAppendix. The FACADE Investigators eTable 1. Inclusion and Exclusion Criteria Used in the FACADE Trial eTable 2. Interventions eTable 3. FACADE Discharge Checklist eTable 4. Schedule of Enrollment, Interventions and Assessments eTable 5. Reasons for Exclusion in 643 Patients eTable 6. Operated Levels and Number of Operated Levels eTable 7. Primary and Secondary Outcomes at Different Time Points eTable 8. Adverse Events, Re-admissions to Hospital and Crossovers (Number of Patients) eTable 9. Sensitivity Analyses: NDI Score in Per-Protocol and As-Treated Analyses eTable 10. Missing Data Items (No. of Patients) [file jamanetwopen-e2447459-s002.pdf]

Supplemental Online Content

Lönnrot K, Taimela S, Satopää J, et al. Ambulatory care vs overnight hospitalization after anterior surgery for cervical radiculopathy: the FACADE randomized clinical trial. *JAMA Netw Open*. 2024;7(11):e2447459. doi:10.1001/jamanetworkopen.2024.47459

eAppendix. The FACADE Investigators

eTable 1. Inclusion and Exclusion Criteria Used in the FACADE Trial

eTable 2. Interventions

eTable 3. FACADE Discharge Checklist

eTable 4. Schedule of Enrollment, Interventions and Assessments

eTable 5. Reasons for Exclusion in 643 Patients

eTable 6. Operated Levels and Number of Operated Levels

eTable 7. Primary and Secondary Outcomes at Different Time Points

eTable 8. Adverse Events, Re-admissions to Hospital and Crossovers (Number of Patients)

eTable 9. Sensitivity Analyses: NDI Score in Per-Protocol and As-Treated Analyses

eTable 10. Missing Data Items (No. of Patients)

This supplemental material has been provided by the authors to give readers additional information about their work.

eAppendix: The FACADE Investigators

Writing Committee
Teppo Järvinen, M.D., Ph.D., Chair, Helsinki University Hospital
Kimmo Lönnrot, M.D., Ph.D., Principal Investigator, Co-Chair, Helsinki University Hospital
Tomasz Czuba, B.S., Ph.D., Trial statistician, University of Gothenburg, Gothenburg, Sweden
Simo Taimela, M.D., Ph.D., Co-Chair, Helsinki University Hospital
Rahul Raj, M.D., Ph.D., Helsinki University Hospital

Steering Committee
Kimmo Lönnrot (chair),
Simo Taimela (co-chair),
Teppo Järvinen (co-chair),
Ville Leinonen (site chair, Oulu)
Ilkka Saarenpää (site chair, Turku)
Pirjo Toivonen (research coordination)

Contributions: All writing committee members have been involved in drafting the manuscript or revising it critically for important intellectual content. All authors read and approved the final manuscript.

Methods Centre (Helsinki University Hospital): Kimmo Lönnrot, Teppo Järvinen, Simo Taimela, Pirjo Toivonen.

Data Safety Monitoring Board: Kari Tikkinen (chair), Christoph Schwartz, Timo Koivisto.

Study Concept and Design: Kimmo Lönnrot, Simo Taimela and Teppo Järvinen.

Statistics: Tomasz Czuba.

Data Management: All the data was centrally collected to electric case report form database (eCRF) via internet access. Consistency of data was supervised by study nurses Maarit Tuominen and Pirjo Toivonen.

Patient Contacts: Maarit Tuominen, Pirjo Toivonen and Marketta Rautanen.

Clinical Investigators:

Helsinki University Hospital

Patient screening and recruitment: Kimmo Lönnrot, Jarno Satopää, Behnam Rezai-Jahromi and Anniina Koski-Palkén.

Surgery*:* Kimmo Lönnrot, Matti Seppälä, Leena Kivipelto, Juha Antinheimo and Miikka Korja.

Trial personnel responsible for assessment of postoperative complications and subsequent randomization: Johannes Förster, Marja Silvasti-Lundell and Mikko Pitkänen.

Turku University Hospital

Patient screening and recruitment*:* Ilkka Saarenpää and Juri Kivelev.

Surgery*:* Juri Kivelev.

© 2024 Lönnrot K et al. *JAMA Network Open*

Trial personnel responsible for assessment of postoperative complications and subsequent randomization: Ilkka Saarenpää and Riitta Westermark.

Oulu University Hospital

Patient screening and recruitment: Ville Leinonen and Mikko Kauppinen.

Surgery: Mikko Kauppinen.

Trial personnel responsible for assessment of postoperative complications and subsequent randomization: Mikko Kauppinen.

© 2024 Lönnrot K et al. *JAMA Network Open*

eTable 1: Inclusion and Exclusion Criteria Used in the FACADE Trial

**Inclusion criteria**
1) Cervical radiculopathy syndrome (CRS) unresponsive to non-operative treatment for at least six weeks or with severe progressive signs and symptoms of nerve root compression during conservative treatment of shorter duration.<br>2) CRS is defined as pain, paresis or paresthesia in corresponding nerve root distribution areas of C5, C6, C7 or C8.<br>3) Nerve root stenosis determined by magnetic resonance imaging at treatment level correlating to CRS/symptoms<br>4) Neck Disability Index score ≥30 out of 100<br>5) Age between 18 to 62 years<br>6) No previous cervical operations<br>7) Currently employed<br>8) No co-morbidities causing a need for a sick leave<br>9) Provision of informed consent from the participant<br>10) No contraindication for randomization in postoperative check (see below)
**Exclusion criteria**
1) MRI finding inconsistent with patient's symptoms<br>2) Diagnosed osteoporosis or permanent use of oral corticosteroids<br>3) ACDF operation requiring plate or cage fixation with screws<br>4) Active malignancy<br>5) American Society of Anesthesiologists Physical Status Classification system (ASA) 4 and 5 patients (seriously ill patients)<br>6) Pregnancy<br>7) Abundant use of alcohol, drugs or narcotics<br>8) No possibility to be accompanied by an adult person over the first postoperative night after the surgery<br>9) Insufficient Finnish language skills<br>10) Distance to the closest hospital emergency more than 60 min

© 2024 Lönnrot K et al. *JAMA Network Open*

eTable 2: Interventions

Overnight hospital surveillance
Patients allocated to hospital surveillance were kept at a hospital ward overnight. The morning after the surgery, a neurosurgeon on duty assessed the patients for complications using the FACADE discharge checklist (eTable 3) prior to discharge.
**Ambulatory care**
A ward nurse evaluated all patients allocated to the outpatient group approximately 6–8 hours after surgery using a standardized FACADE discharge checklist (eTable 3). If the patient fulfilled all discharge criteria, he/she was informed on how to deal with any concerns and discharged.

© 2024 Lönnrot K et al. *JAMA Network Open*

eTable 3: FACADE Discharge Checklist

**The following checklist items must be approved by study personnel before the patient is discharged:**
1) Operating surgeon agrees, that there is no reason for excluding the patient form randomisation<br>2) No new neurological deficit or intolerable pain<br>3) No swelling nor hemorrhage on neck<br>4) Normal ability to swallow (must be checked with small amount of water)<br>5) Walking and urination are independent<br>6) Patient is accompanied at home

eTable 4: Schedule of Enrollment, Interventions and Assessments

	Study period							
	**Enrolment**	**Allocation**	**Post-allocation**					
**Timepoint**	Baseline	Surgery	1-wk	2-wk	3-wk	1-mo	3-mo	6-mo
**Endollement:**								
Eligibility screen	x							
Informed consent	x							
Allocation		x						
MRI	x							
**Interventions:**								
Overnight surveillance		x						
Ambulatory care		x						
**Assessments:**								
Adverse events		x	x	(x)	(x)	x	x	x
Work Ability Score (WAS)	x		x	(x)	(x)	x	x	x
Return to work			x	(x)	(x)	x	x	x
Return to previous activities			x	(x)	(x)	x	x	x
Operative success*			x	(x)	(x)	x	x	x
Satisfaction to the treatment								x
Health resource utilization			x	(x)	(x)	x	x	x
Clinical examination	x					(x)		(x)
NDI	x					x	x	x
NRS-AP	x		x	(x)	(x)	x	x	x
NRS-NP	x		x	(x)	(x)	x	x	x
EQ-5D-5L	x							x

() if required.
mo, month(s) postoperatively;
wk, week(s) postoperatively;
NDI, Neck Disability Index;
NRS-AP, Numeric Rating Scale for Arm Pain;
NRS-NP, Numeric Rating Scale for Neck Pain;
*Odom's rating scale, in which the patient subjectively rates the perception of operative success from poor to excellent.
EQ-5D-5L, a standardized health-related quality of life instrument for assessing a patient's general health and treatment outcome and details a patient's self-assessed health profile.

© 2024 Lönnrot K et al. *JAMA Network Open*

eTable 5: Reasons for Exclusion in 643 Patients

Reason for exclusion[a]	No. of patients
Age (under 18 or over 62 years)	172
Myelopathy	163
Previous cervical spine surgery	107
Co-morbidities causing a need for an extended sick leave	104
Neck Disability Index score lower than 30 (out of 100)	79
Currently unemployed	58
Unproficiency in Finnish language	56
Distance to hospital (>1h)	48
Duration of symptoms less than six weeks	43
ASA 4 and 5 patients (seriously ill patients)	9
Diagnosed osteoporosis or permanent use of oral corticosteroids	7
Impaired ability to cooperate (substance abuse, mental disorder, dementia)	7
No accompanying adult for the first postoperative night after the surgery	5
Active malignancy	2
Pregnancy	1
**Total**	**861**

[a]Some of the patients had more than one reason for exclusion.

© 2024 Lönnrot K et al. *JAMA Network Open*

eTable 6: Operated Levels and Number of Operated Levels

Variable	Ambulatory care (n=52)	Overnight hospital surveillance (n=53)
**Level of ACDF[a] (all levels operated)**	70	61
C4–5	2/70 (3%)	2/61 (3%)
C5–6	38/70 (54%)	22/61 (36%)
C6–7	29/70 (42%)	36/61 (59%)
C7–Th1	1/70 (1%)	1/61 (2%)
**One level ACDF[a]**	34	45
C4–5	0/34 (0%)	0/45 (0%)
C5–6	20/34 (59%)	14/45 (31%)
C6–7	13/34 (38%)	30/45 (67%)
C7–Th1	1/34 (3%)	1/45 (2%)
**Two level ACDF[a]**	18	8
C4–5–6	2/18 (11%)	2/8 (25%)
C5–6–7	16/18 (89%)	6/8 (75%)

[a] One patient could have been operated for one or two levels (total number of levels operated on reported and percentages calculated according to total levels).
[b] Patient-level information (percentages calculated according to number patients).

© 2024 Lönnrot K et al. *JAMA Network Open*

eTable 7: Primary and Secondary Outcomes at Different Time Points[a]

Outcomes	Ambulatory care (N=52) mean (95% CI)	Overnight hospital surveillance (N=53) mean (95% CI)	Between-group mean difference Ambulatory care - Overnight hospital surveillance (95% CI)
**1 week**			
Primary outcome			
NDI score[b], %	NA	NA	NA
Secondary outcome	N=51	N=51	
Arm Pain[c]	2.3 (1.7 to 3.0)	2.2 (1.6 to 2.8)	0.11 (-0.70 to 0.93)
Neck Pain[c]	4.6 (4.0 to 5.2)	4.5 (3.9 to 5.1)	0.17 (-0.69 to 1.0)
Return to previous activities[d], %	8 (0 to 15)	20 (9 to 31)	-12 (-25 to 1)
Return to work[e], %	13 (4 to 23)	15 (6 to 25)	-1.9 (-15 to 11)
Work ability Score[f]	4.0 (3.4 to 4.7)	4.2 (3.6 to 4.7)	-0.2 (-1.1 to 0.71)
EQ-5D-5L (Utility score)[g]	NA	NA	NA
Patient satisfaction[h]	NA	NA	NA
Operative success ODOM[i], %	58 (45 to 72)	74 (62 to 86)	-15 (-33 to 3)
Postoperative dysphonia[j]	2.3 (1.8 to 2.8)	1.7 (1.2 to 2.2)	0.59 (-0.10 to 1.3)
Postoperative dysphagia[j]	3.5 (3.1 to 3.8)	2.1 (1.7 to 2.5)	1.3 (0.79 to 1.9)
**2 weeks**			
Primary outcome			
NDI score[b], %	NA	NA	NA
Secondary outcome	N=43	N=44	
Arm Pain[c]	2.4 (1.8 to 3.0)	1.9 (1.3 to 2.5)	0.5 (-0.4 to 1.3)
Neck Pain[c]	4.1 (3.4 to 4.7)	3.8 (3.4 to 4.7)	0.3 (-0.6 to 1.2)
Return to previous activities[d], %	21 (9 to 33)	30 (16 to 43)	-8.6 (-27 to 10)
Return to work[e], %	34 (22 to 48)	29 (17 to 41)	5.7 (-12 to 24)
Work ability Score[f]	5.3 (4.6 to 5.9)	4.8 (4.1 to 5.5)	0.5 (-0.5 to 1.4)
EQ-5D-5L (Utility score)[g]	NA	NA	NA
Patient satisfaction[h]	NA	NA	NA
Operative success ODOM[i], %	56 (39 to 73)	69 (58 to 86)	-13 (-37 to 11)
Postoperative dysphonia[j]	1.5 (1.0 to 2.0)	0.9 (0.4 to 1.4)	0.6 (-0.1 to 1.3)
Postoperative dysphagia[j]	1.7 (1.3 to 2.1)	1.3 (0.9 to 1.7)	0.4 (-0.2 to 0.9)
**3 weeks**			
Primary outcome			
NDI score[b], %	NA	NA	NA
Secondary outcome	N=33	N=35	
Arm Pain[c]	2.1 (1.4 to 2.8)	1.6 (0.9 to 2.3)	0.5 (-0.4 to 1.4)
Neck Pain[c]	3.1 (2.4 to 3.8)	3.4 (2.7 to 4.1)	-0.3 (-1.3 to 0.6)
Return to previous activities[d], %	24 (10 to 39)	38 (22 to 55)	-14 (-36 to 8)
Return to work[e], %	56 (42 to 69)	50 (36 to 64)	5.7 (-13 to 25)
Work ability Score[f]	6.0 (5.2 to 6.7)	5.7 (4.9 to 6.4)	0.3 (-0.7 to 1.4)
EQ-5D-5L (Utility score)[g]	NA	NA	NA
Patient satisfaction[h]	NA	NA	NA
Operative success ODOM[i], %	68 (50 to 86)	73 (56 to 90)	-5 (-30 to 20)
Postoperative dysphonia[j]	1.0 (0.4 to 1.5)	0.6 (0.1 to 1.2)	0.4 (-0.4 to 1.2)
Postoperative dysphagia[j]	1.1 (0.6 to 1.5)	0.8 (0.3 to 1.2)	0.3 (-0.3 to 0.9)
**1 month**			
Primary outcome	N=51	N=46	
NDI score[b], %	21 (17 to 24)	19 (15 to 23)	1.8 (-3.8 to 7.5)
Secondary outcome	N=51	N=47	
Arm Pain[c]	1.9 (1.3 to 2.4)	1.4 (0.8 to 2.0)	0.5 (-0.4 to 1.3)
Neck Pain[c]	2.2 (1.6 to 2.8)	2.2 (1.6 to 2.8)	0.0 (-0.9 to 0.9)
Return to previous activities[d], %	63 (49 to 76)	61 (47 to 75)	1.8 (-17 to 21)
Return to work[e], %	65 (52 to 78)	69 (57 to 82)	-3.8 (-22 to 14)

© 2024 Lönnrot K et al. *JAMA Network Open*

Work ability Score[f]	6.8 (6.2 to 7.4)	7.1 (6.4 to 7.7)	-0.3 (-1.2 to 0.6)
EQ-5D-5L (Utility score)[g]	NA	NA	NA
Patient satisfaction[h]	NA	NA	NA
Operative success ODOM[i], %	73 (60 to 85)	78 (66 to 90)	-5.7 (-23 to 11)
Postoperative dysphonia[j]	0.9 (0.4 to 1.3)	0.6 (0.1 to 1.3)	0.3 (-0.4 to 1.0)
Postoperative dysphagia[j]	0.7 (0.3 to 1.1)	0.5 (0.1 to 0.9)	0.2 (-0.4 to 0.8)
**3 months**			
Primary outcome	N=40	N=42	
NDI score[b], %	16 (12 to 20)	13 (9.3 to 17)	2.5 (-3.5 to 8.4)
Secondary outcome	N=40	N=42	
Arm Pain[c]	1.7 (1.1 to 2.4)	1.2 (0.6 to 1.8)	0.5 (-0.3 to 1.4)
Neck Pain[c]	2.5 (1.9 to 3.2)	1.7 (1.1 to 2.4)	0.8 (-0.12 to 1.7)
Return to previous activities[d], %	85 (74 to 96)	88 (78 to 98)	-3 (-18 to 12)
Return to work[e], %	63 (49 to 78)	74 (61 to 87)	-11 (-31 to 9)
Work ability Score[f]	7.2 (6.5 to 7.9)	8.3 (7.7 to 9.0)	-1.2 (-2.1 to -0.2)
EQ-5D-5L (Utility score)[g]	NA	NA	NA
Patient satisfaction[h]	NA	NA	NA
Operative success ODOM[i], %	60 (41 to 79)	92 (81 to 100)	-31 (-54 to -10)
Postoperative dysphonia[j]	0.7 (0.2 to 1.3)	0.4 (-0.1 to 0.9)	0.3 (-0.4 to 1.1)
Postoperative dysphagia[j]	0.2 (-0.2 to 0.7)	0.1 (-0.3 to 0.5)	0.1 (-0.5 to 0.7)
**6 months**			
Primary outcome	N=47	N=48	
NDI score[b], %	13 (9 to 17)	12 (8 to 16)	1.1 (-4.6 to 6.8)
Secondary outcome	N=38	N=39	
Arm Pain[c]	1.7 (1.0 to 2.3)	1.6 (0.9 to 2.2)	0.1 (-0.8 to 1)
Neck Pain[c]	2.1 (1.5 to 2.8)	1.8 (1.2 to 2.5)	0.3 (-0.6 to 1.2)
Return to previous activities[d], %	82 (69 to 94)	92 (84 to 100)	-10 (-26 to 4)
Return to work[e], %	89 (79 to 99)	92 (84 to 100)	-2.9 (-16 to 10)
Work ability Score[f]	7.5 (6.8 to 8.3)	7.8 (7.1 to 8.6)	-0.3 (-1.3 to 0.7)
EQ-5D-5L (Utility score)[g]	0.94 (0.91 to 0.96)	0.94 (0.92 to 0.96)	0.004 (-0.027 to 0.034)
Patient satisfaction[h]	97 (83 to 99)	95 (81 to 97)	3 (-6 to 11)
Operative success ODOM[i], %	82 (69 to 94)	82 (69 to 94)	0 (-17 to 17)
Postoperative dysphonia[j]	0.7 (0.1 to 1.2)	0.5 (-0.04 to 1.0)	1.7 (-0.6 to 0.9)
Postoperative dysphagia[j]	0.3 (-0.1 to 0.8)	0.1 (-0.3 to 0.5)	0.2 (-0.4 to 0.8)

[a] The point estimates are derived from the Repeated measures mixed model using all available data.
[b] Neck Disability Index (NDI) score is a widely used and validated patient reported tool assessing neck pain and radiculopathy related discomfort and symptoms in daily life. The instrument consists of 10 items. In each item, the range of the score is from 0 (no disability) to 5 (extreme disability). We used the percentage of theoretical maximum score.
[c] Arm and neck pain were reported on 0-10 numerical rating scale where 0 is no pain and 10 is the worst imaginable pain.
[d] Patients were asked if they were ready to return to leisure activity they had preoperatively named being impaired by cervical radiculopathy syndrome. Responses were given as “Yes” or “No”.
[e] Patients were asked if they were able to return to work. Responses were given as “Yes” or “No”.
[f] Work ability score is an 11-point NRS in which a patient will assess his or her current work ability compared with the lifetime best, with a possible score of 0 (‘completely unable to work’) to 10 (‘work ability at its best’).
[g] EQ-5D-5L is a standardized health-related quality of life instrument for assessing a patient’s general health and treatment outcome and details a patient’s self-assessed health profile. The scores can be converted into quality-adjusted life years (QALYs).
[h] Patients’ global assessment of satisfaction to the treatment at 6 months after operation with this question: “If you were to choose again, would you choose an operative treatment?” Responses were given as “Yes” or “No”.
[i] Modified Odom’s criteria, in which the patient subjectively rates the perception of operative success from poor to excellent. We considered the first and second categories (‘excellent’ and ‘good’) as a successful outcome of the operation and, conversely, last two categories (‘fair’ and ‘poor’) as an unsuccessful outcome.
[j] Postoperative dysphonia and dysphagia will be assessed with 0 to 10 numerical rating scales NRS-DP (NRS-dysphonia) and NRS-OP (NRS-dysphagia) at each postoperative time point. NRS will be assessed on an 11-unit scale ranging from 0 (no dysphonia/dysphagia) to 10 (extreme dysphonia/dysphagia).

© 2024 Lönnrot K et al. *JAMA Network Open*

eTable 8: Adverse Events, Re-admissions to Hospital and Crossovers (Number of Patients)[a]

Description	Ambulatory care (N=52)	Overnight hospital surveillance (N=53)
**Serious adverse event** [b]		
Deep venous thrombosis in leg	0	1
**Minor adverse event** [b]		
Local infection	3	0
Transient dysphagia	2	0
Panic disorder	1	0
Foraminal re-stenosis	1	0
**Rehospitalizations (<30 days)**	2	0
**Crossovers**		
Postoperative neck pain and nausea	2	0
Wound surveillance	1	0
Urinary retention	1	0

[a] Each patient had only one adverse event.

[b] Acute care revisit

© 2024 Lönnrot K et al. *JAMA Network Open*

eTable 9: Sensitivity Analyses: NDI Score in Per-Protocol[a] and As-Treated[b] Analyses

	Per Protocol			As-treated		
**NDI score**	**Ambulatory care mean (95% CI)**	**Overnight hospital surveillance mean (95% CI)**	**Between-group mean difference (95% CI)**	**Randomized to Ambulatory care mean (95% CI)**	**Randomized to Overnight hospital surveillance mean (95% CI)**	**Between-group mean difference (95% CI)**
**1 month**	21 (17 to 25)	19 (16 to 23)	2.1 (-3.8 to 8.0)	21 (17 to 25)	19 (15 to 22)	2.3 (-3.4 to 8.0)
**3 months**	16 (12 to 21)	13 (9 to 18)	2.8 (-3.3 to 8.9)	16 (12 to 21)	13 (9 to 17)	2.9 (-3.0 to 8.9)
**6 months**	13 (9 to 18)	12 (8 to 16)	1.2 (-4.7 to 7.1)	13 (9 to 18)	12 (8 to 16)	1.3 (-4.5 to 7.0)

[a] In Per Protocol analysis were analyzed the overnight surveillance group and the patients in the ambulatory care group who adhered to the assigned treatment protocol (were discharged on the day of surgery).

[b] In as-treated analysis groups were analyzed as they actually received care at the different follow-up time points. The number of patients in Overnight hospital surveillance group increased in subsequent follow-up points because of crossovers (n=4) from ambulatory care to overnight surveillance.

© 2024 Lönnrot K et al. *JAMA Network Open*

eTable 10: Missing Data Items (No. of Patients)

	Baseline		1 week		1 month		6 months	
**Outcome**	**Ambulatory care**	**Overnight hospital surveillance**	**Ambulatory care**	**Overnight hospital surveillance**	**Ambulatory care**	**Overnight hospital surveillance**	**Ambulatory care**	**Overnight hospital surveillance**
**NDI**	0	0	NA	NA	1	6	5	5
**WAS**	0	0	1	2	1	6	14	14
**Return to work**	0	0	0	0	0	0	14	14
**Return to previous activities**	NA	NA	1	2	1	6	14	14
**ODOM**	NA	NA	1	2	1	6	14	14
**Satisfaction to treatment**	NA	NA	NA	NA	NA	NA	14	14
**NRS-AP**	0	0	1	2	1	6	14	14
**NRS-NP**	0	1	1	2	1	6	14	14
**EQ-5D-5L**	0	0	NA	NA	NA	NA	14	14

NDI, Neck Disability Index;
WAS, Work Ability Score;
ODOM, Odom's rating scale;
NRS-AP, Numeric Rating Scale for Arm Pain;
NRS-NP, Numeric Rating Scale for Neck Pain;
EQ-5D-5L, a standardized health-related quality of life instrument for assessing a patient's general health and treatment outcome and details a patient's self-assessed health profile.

© 2024 Lönnrot K et al. *JAMA Network Open*
