# Supplementary material for: Ambulatory Care vs Overnight Hospitalization After Anterior Surgery for Cervical Radiculopathy: The FACADE Randomized Clinical Trial
Source: JAMA Netw Open. 2024 Nov 27;7(11):e2447459. doi: 10.1001/jamanetworkopen.2024.47459 (PMC12124692; doi:10.1001/jamanetworkopen.2024.47459)
Supplement: Supplement 3. — Data Sharing Statement [file jamanetwopen-e2447459-s003.pdf]

Data Sharing Statement

Lönnrot. Ambulatory Care vs Overnight Hospitalization After Anterior Surgery for Cervical Radiculopathy. *JAMA Netw Open*. Published November 27, 2024.
doi:10.1001/jamanetworkopen.2024.47459

Data
Additional Information: ClinicalTrials.gov ID NCT03979443,
https://www.clinicaltrials.gov/study/NCT03979443
Data available: Yes
Data types: Deidentified participant data
How to access data: All data requests should be submitted to the corresponding authors for consideration. Access to anonymized data may be granted following review and consideration of the Act on the Secondary Use of Health and Social Data in Finland.
When available: With publication

Supporting Documents
Document types: Statistical/analytic code
How to access documents: All data requests should be submitted to the corresponding authors for consideration. Access to anonymized data may be granted following review and consideration of the Act on the Secondary Use of Health and Social Data in Finland.
When available: With publication

Additional Information
Who can access the data: All data requests should be submitted to the corresponding authors for consideration. Access to anonymized data may be granted following review and consideration of the Act on the Secondary Use of Health and Social Data in Finland.
Types of analyses: Individual-data meta-analysis
Mechanisms of data availability: All data requests should be submitted to the corresponding authors for consideration. Access to anonymized data may be granted following review and consideration of the Act on the Secondary Use of Health and Social Data in Finland.
Any additional restrictions: Act on the Secondary Use of Health and Social Data in Finland.
